# Systemic Inflammatory Markers, Thyroid Autoimmunity, and Neuroretinal Structure in Graves’ Disease: A Comparative OCT Study According to Ophthalmopathy Status

**DOI:** 10.3390/metabo16100782

**Published:** 2026-10-16

**Authors:** Sinem Keser, Aykut Bulu

**Affiliations:** 1 Department of Ophthalmology, Fethi Sekin City Hospital, Elazığ 23280, Turkey; 2 Department of Internal Medicine, Fethi Sekin City Hospital, Elazığ 23280, Turkey; abulu@firat.edu.tr

**Keywords:** Graves’ disease, optical coherence tomography, retinal nerve fiber layer, autoimmunity, inflammation

## Abstract

**Background:** Graves’ disease is a systemic autoimmune disorder that may affect ocular structures even in the absence of clinically apparent ophthalmopathy. Optical coherence tomography (OCT) enables objective assessment of neuroretinal structures, but the relationships between OCT findings, systemic inflammatory indices, thyroid hormone levels, and thyroid autoimmunity markers in Graves’ disease remain incompletely defined. This study aimed to evaluate the associations between OCT-derived neuroretinal measurements and systemic inflammatory, hormonal, and autoimmune markers according to ophthalmopathy status. **Methods:** This retrospective cross-sectional study included 161 participants: 60 healthy controls, 50 Graves’ disease patients with ophthalmopathy (GO+), and 51 Graves’ disease patients without ophthalmopathy (GO−). Central macular thickness (CMT) and circumpapillary retinal nerve fiber layer (cpRNFL) thickness measurements were obtained using spectral-domain OCT. Macular RNFL (mRNFL) thickness was not evaluated. Laboratory evaluation included complete blood count, glucose, urea, creatinine, aspartate aminotransferase (AST), alanine aminotransferase (ALT), total cholesterol, high-density lipoprotein cholesterol (HDL-C), low-density lipoprotein cholesterol (LDL-C), triglycerides, C-reactive protein (CRP), thyroid-stimulating hormone (TSH), free triiodothyronine (fT3), free thyroxine (fT4), anti-thyroid peroxidase (anti-TPO), and anti-thyroglobulin (anti-Tg) antibodies. **Results:** In the primary three-group OCT analysis, significant differences were observed for superior (*p* = 0.010, *q* = 0.0125), inferior (*p* < 0.001, *q* = 0.0024), temporal (*p* = 0.002, *q* = 0.0050), and nasal cpRNFL thickness (*p* = 0.006, *q* = 0.0092), and all remained significant after FDR correction; CMT did not differ among the groups (*p* = 0.925, *q* = 0.925). The lowest cpRNFL thickness values were observed in the GO− subgroup, particularly in the inferior quadrant (116.39 ± 27.52 vs. 136.65 ± 20.01 µm, post hoc *p* < 0.001); however, this finding should be interpreted cautiously because standardized GO activity and severity staging was not consistently available. Systemic inflammatory indices were largely similar between groups, whereas CRP levels were higher in Graves’ disease in the exploratory analysis (3.87 ± 3.40 vs. 1.86 ± 1.11 mg/L, nominal *p* < 0.001). Several weak correlations between cpRNFL parameters and fT4, CRP, and anti-TPO reached nominal significance; however, none remained statistically significant after FDR correction. In multivariable analysis restricted to patients with Graves’ disease, GO+ status remained associated with greater inferior cpRNFL thickness compared with GO− status, whereas anti-TPO was not associated with inferior cpRNFL thickness after adjustment. **Conclusions:** Sectoral cpRNFL differences were observed among the control, GO+, and GO− groups, whereas CMT remained comparable. However, the lower cpRNFL measurements in patients classified as GO− should be interpreted cautiously because of retrospective ophthalmopathy classification, potential misclassification, differential exclusion rates, and residual confounding. These findings are strictly exploratory and hypothesis-generating and require prospective validation. Biomarker–OCT correlations did not remain statistically significant after FDR correction.

## 1. Introduction

Graves’ disease is the most common cause of thyrotoxicosis and a systemic autoimmune disorder characterized not only by thyroid dysfunction but also by involvement of extrathyroidal tissues [1]. The clinical manifestations of the disease extend beyond excessive thyroid hormone production and reflect a complex interaction of immune dysregulation, inflammatory activation, and tissue-specific responses. Therefore, contemporary management of Graves’ disease requires not only restoration of euthyroidism but also careful evaluation of extrathyroidal involvement [2]. 

Graves’ ophthalmopathy (GO) represents the most characteristic extrathyroidal manifestation of Graves’ disease and is a multifactorial inflammatory condition involving orbital fibroblast activation, cytokine-mediated immune responses, adipogenesis, and remodeling of orbital connective tissues [3,4,5]. Although GO is considered the defining ocular manifestation of the disease, increasing evidence suggests that ocular involvement may not be limited to patients with clinically apparent ophthalmopathy. Structural and microvascular alterations may also occur in patients without obvious orbital signs, indicating that neuro-ophthalmic involvement may begin at earlier or subclinical stages of the disease [6,7]. 

The retina and the retinal nerve fiber layer (RNFL) are highly metabolically active structures that are particularly vulnerable to inflammatory, autoimmune, and microvascular disturbances. Because these tissues depend on tightly regulated perfusion and cellular homeostasis, they may be affected by systemic immune-mediated processes even in the absence of advanced orbital disease. For this reason, evaluation of neuroretinal structures may provide additional insight into ocular involvement in Graves’ disease beyond conventional clinical assessment of ophthalmopathy [7,8]. 

Optical coherence tomography (OCT) enables objective and reproducible measurement of neuroretinal structures through high-resolution, non-invasive imaging, allowing quantitative assessment of macular thickness and circumpapillary retinal nerve fiber layer (cpRNFL) parameters [7,8]. Previous studies in the spectrum of thyroid eye disease have demonstrated that alterations in RNFL thickness, ganglion cell complex measurements, optic nerve head perfusion, and peripapillary microvascular density may be associated with disease stage, activity, or optic nerve involvement [7,8]. These findings support the concept that neuroretinal changes may develop not only in advanced ophthalmic disease but also in earlier or clinically less apparent phases of Graves’ disease. 

In addition to local orbital mechanisms, systemic inflammatory activity and thyroid autoimmunity may contribute to extrathyroidal manifestations. Hematologic indices derived from routine complete blood count parameters, including the neutrophil-to-lymphocyte ratio (NLR), platelet-to-lymphocyte ratio (PLR), monocyte-to-lymphocyte ratio (MLR), and systemic immune-inflammation index (SII), have been widely used as accessible markers of systemic inflammatory burden in autoimmune disorders [9,10]. When evaluated together with thyroid function tests and thyroid autoantibodies, particularly anti-thyroid peroxidase antibody (anti-TPO) and anti-thyroglobulin antibody (anti-Tg), these parameters may provide a broader view of systemic immune-metabolic status in Graves’ disease [9,10]. The rationale for evaluating these indices in relation to neuroretinal structure is based on the potential contribution of systemic immune-inflammatory activity to microvascular and neuroaxonal injury. NLR has been proposed to reflect the balance between innate inflammatory activation and adaptive immune regulation, PLR may reflect platelet-related inflammatory and vascular responses, MLR represents monocyte-mediated inflammatory activity relative to lymphocyte-mediated immunity, and SII integrates neutrophil, platelet, and lymphocyte counts as a composite measure of systemic inflammation. Because the retinal ganglion cell axons forming the cpRNFL are metabolically active and potentially vulnerable to inflammatory and microvascular disturbances, these readily available indices may provide indirect markers of the systemic inflammatory milieu associated with neuroretinal structural alterations. However, they are nonspecific systemic markers and should not be regarded as direct measures of localized retinal inflammation.

However, although previous studies have investigated OCT findings in thyroid eye disease and others have evaluated systemic inflammatory indices or thyroid autoimmunity separately, studies integrating complete blood count–derived inflammatory markers, thyroid autoimmunity parameters, and OCT-based neuroretinal measurements within the same cohort of Graves’ disease patients stratified according to the presence or absence of Graves’ ophthalmopathy remain scarce. In particular, comparative analyses including Graves’ disease patients with ophthalmopathy (GO+), Graves’ disease patients without ophthalmopathy (GO−), and healthy controls within a single analytical framework are limited. 

Therefore, the present study aimed to investigate the relationships between systemic inflammatory indices, thyroid function parameters, thyroid autoimmunity markers, and OCT-derived neuroretinal measurements in patients with Graves’ disease, and to determine whether these associations differ according to ophthalmopathy status. The primary outcome was the comparison of macular thickness and cpRNFL parameters among GO+, GO−, and healthy control groups. The secondary outcome was to evaluate the associations between OCT parameters and systemic inflammatory indices as well as thyroid autoimmunity markers. Through this integrated approach, we aimed to determine whether neuroretinal differences may be present in patients classified as GO− based on available clinical records and to provide additional data on neuroretinal involvement in Graves’ disease.

## 2. Materials and Methods

### 2.1. Study Design and Participants

This retrospective cross-sectional comparative study was conducted at the Departments of Internal Medicine and Ophthalmology of Fethi Sekin City Hospital between 1 January 2020 and 1 January 2026. The study protocol was approved by the Non-Interventional Clinical Research Ethics Committee of Fethi Sekin City Hospital (decision number: 2026/24-07, date: 19 February 2026), and the study was performed in accordance with the principles of the Declaration of Helsinki. The study period (1 January 2020–1 January 2026) refers exclusively to the period during which the clinical, laboratory, and OCT data had been routinely recorded in the hospital database. Data extraction for research purposes and all subsequent statistical analyses were initiated only after ethics committee approval had been obtained on 19 February 2026. Because the study was retrospective and based exclusively on previously recorded hospital data, the requirement for written informed consent was waived by the ethics committee.

The study population consisted of patients with Graves’ disease and healthy control subjects who had undergone ophthalmologic examination and OCT evaluation during the study period. Patients with Graves’ disease were classified according to documented ophthalmopathy status (GO+ and GO−). Healthy control candidates were retrospectively selected from individuals who attended the same ophthalmology outpatient clinic during the same study period and had undergone ophthalmologic examination and OCT imaging. Control subjects had no known thyroid disease or autoimmune disorder, had normal findings on ophthalmologic examination, and met the same applicable ocular and systemic eligibility criteria as the Graves’ disease groups.

Clinical data, laboratory parameters, and OCT measurements were obtained retrospectively from the electronic hospital database. Hematological parameters, systemic inflammatory indices, thyroid function tests, thyroid autoimmunity markers, macular thickness, and RNFL measurements were compared between groups. The primary comparative analysis was performed among the control, GO+, and GO− groups. A pooled comparison of all patients with Graves’ disease versus controls was additionally performed as a secondary/exploratory analysis to provide a complementary overall disease-level comparison.

Only subjects with available laboratory data and OCT measurements obtained within a maximum interval of 10 days were included in the analysis.

### 2.2. Diagnosis of Graves’ Disease

Patients were included in the Graves’ disease groups if an established clinical diagnosis of Graves’ disease made by the treating physician was documented in the medical records. The diagnosis had been made in routine clinical practice on the basis of the overall clinical, biochemical, and, when available, immunological and imaging findings, in accordance with accepted diagnostic principles [1,2]. These findings could include clinical manifestations of hyperthyroidism, biochemical thyrotoxicosis characterized by suppressed TSH with elevated fT4 and/or fT3 levels, TRAb positivity, and diffuse goiter on physical examination or thyroid ultrasonography. The “at least two criteria” rule was not used as a predefined or validated diagnostic algorithm for the present study. Because TRAb measurements were not available for all patients, TRAb positivity was not required for study inclusion.

### 2.3. Diagnosis of Graves’ Ophthalmopathy

The presence of ophthalmopathy was determined retrospectively from detailed ophthalmologic examination records, with reference to the clinical features described in the European Group on Graves’ Orbitopathy (EUGOGO) guidelines [3]. Patients were classified as GO+ when one or more ocular findings considered compatible with Graves’ ophthalmopathy were documented by the examining ophthalmologist, including proptosis, eyelid retraction or edema, conjunctival hyperemia or chemosis, limitation of extraocular muscle movement, or diplopia. These findings were interpreted in the context of the established diagnosis of Graves’ disease and the overall ophthalmologic examination rather than as isolated signs. Patients with ocular, orbital, or neurologic conditions that could independently affect the optic nerve or retinal structure or mimic Graves’ ophthalmopathy were excluded. These conditions included glaucoma, optic neuropathy, retinal vascular disorders, macular disease, uveitis, diabetic retinopathy, orbital tumors, idiopathic orbital inflammation, myasthenia gravis, cranial nerve palsy, significant refractive error (spherical equivalent > +3.0 D or <−3.0 D), and previous intraocular surgery.

Because of the retrospective design, standardized assessments of GO activity and severity, including the Clinical Activity Score (CAS) and EUGOGO severity grading, were not consistently available for all patients. Objective exophthalmometric thresholds and orbital imaging data were also not systematically available and therefore could not be incorporated into a uniform GO classification algorithm. Consequently, GO status was analyzed as a dichotomous clinical variable based on the ophthalmologic documentation available in the medical records, and some degree of misclassification between the GO+ and GO− groups cannot be excluded.

For the purposes of this study, GO+/GO− status was assigned retrospectively by an ophthalmologist based on the available clinical ophthalmologic records, without access to the OCT measurements used in the study analyses. Thus, the examiner assigning GO status was masked to the OCT results.

### 2.4. Inclusion Criteria

Subjects were eligible for inclusion if they met the following criteria: age ≥ 18 years, confirmed diagnosis of Graves’ disease for the patient groups, absence of known thyroid or autoimmune disease in the control group, availability of both OCT measurements and laboratory data, and a maximum interval of 10 days between OCT examination and blood sampling.

### 2.5. Exclusion Criteria

Subjects were excluded if any of the following conditions were present: diabetes mellitus; hypertension; chronic kidney disease; neurologic disease potentially affecting the RNFL; glaucoma; optic neuropathy, including clinically diagnosed dysthyroid optic neuropathy (DON); retinal vascular disorders; macular disease; uveitis; diabetic retinopathy; orbital tumors; idiopathic orbital inflammation; myasthenia gravis; cranial nerve palsy; significant refractive error (spherical equivalent > +3.0 D or <−3.0 D); previous intraocular surgery; active infection; malignancy; systemic inflammatory disease; or poor-quality OCT images, defined as an image-quality score < 7, motion artifacts, decentration, incomplete acquisition, or segmentation errors. Subjects with incomplete laboratory or OCT data or an interval > 10 days between OCT examination and blood sampling were also excluded.

### 2.6. Participant Selection

A total of 245 potentially eligible subjects with available OCT examinations were identified from hospital records between 1 January 2020 and 1 January 2026. These comprised 165 patients with Graves’ disease, including 71 patients with documented clinical ophthalmopathy (GO+) and 94 patients without documented clinical ophthalmopathy (GO−), and 80 healthy control candidates. Of the 245 potentially eligible subjects, 84 were excluded because of diabetes mellitus (*n* = 15), hypertension (*n* = 18), chronic kidney disease (*n* = 6), neurologic disease potentially affecting the RNFL (*n* = 4), ocular exclusion criteria (*n* = 16), significant refractive error (*n* = 7), poor-quality OCT images or segmentation/acquisition problems (*n* = 8), or incomplete laboratory/OCT data (*n* = 4), or an interval >10 days between OCT examination and blood sampling (*n* = 6). After application of the eligibility criteria, 161 participants were included in the final analysis, comprising 60 healthy controls and 101 patients with Graves’ disease. Among the patients with Graves’ disease included in the final analysis, 50 had documented clinical ophthalmopathy (GO+) and 51 had no documented clinical ophthalmopathy (GO−). Because the study was retrospective, the specific reasons for exclusion could not be reliably reconstructed separately for the control, GO+, and GO− candidate groups from the available records. Therefore, although the overall reasons for exclusion are reported above, group-specific exclusion counts by reason could not be provided. Notably, the proportion of potentially eligible subjects excluded before the final analysis was greater in the GO− group than in the control and GO+ groups. This unequal attrition may have introduced differential selection bias and should be considered when interpreting comparisons involving the GO− group. The participant selection process is summarized in Figure 1.

### 2.7. OCT Measurements

All participants underwent a routine ophthalmologic examination, including assessment of best-corrected visual acuity (BCVA), refractive status, and intraocular pressure (IOP). For the purposes of this study, normal visual acuity was defined as a BCVA of 20/20 (1.0 decimal equivalent) or better, and IOP within the normal range was defined as 10–21 mmHg. Only eyes meeting these criteria were included in the analysis. Participants with a spherical equivalent > +3.0 D or <−3.0 D were excluded. Axial length measurements were not routinely available because of the retrospective study design.

All OCT examinations were performed using a spectral-domain optical coherence tomography device (OCT-HS100, Canon Inc., Tokyo, Japan) under standardized clinical conditions and with the same scanning protocol for all participants. Measurements were obtained after pharmacologic pupil dilation and were performed by experienced ophthalmologists with at least five years of clinical practice in OCT imaging. Because of the retrospective nature and the six-year study period, the exact number of ophthalmologists involved in OCT acquisition could not be reliably determined from the available records. Formal inter- and intra-rater reliability assessments, including intraclass correlation coefficient (ICC) analyses, were therefore not available. Nevertheless, the use of the same OCT device and standardized scanning protocol throughout the study period, together with visual review of automated segmentation for all included scans, was intended to minimize measurement variability.

Central macular thickness (CMT) was assessed using the standard macular scanning protocol of the device (Figure 2). Macular RNFL (mRNFL) thickness was not evaluated because mRNFL measurements were not routinely available in the retrospective OCT records used for this study. cpRNFL thickness was assessed using the device-specific optic disc scanning protocol of the Canon OCT-HS100 (software version 4.2.1). RNFL thickness was measured along a 3.45 mm diameter circular measurement path centered on the optic disc, and the superior, inferior, temporal, and nasal quadrants were analyzed separately (Figure 3). RNFL boundaries were determined using the device’s automated segmentation algorithm. Automated segmentation was visually reviewed for all included scans. No manual correction of segmentation boundaries was performed; scans with segmentation errors affecting the peripapillary RNFL boundaries were excluded from the analysis. OCT scans were evaluated by ophthalmologists who were masked to clinical group classification. A conventional global/average cpRNFL thickness parameter was also not consistently available in the archived OCT records and was therefore not included in the analysis. Consequently, cpRNFL analysis was restricted to the superior, inferior, temporal, and nasal sectoral measurements that were consistently available across the study population.

OCT image quality was assessed using the image-quality indicator provided by the device software, which ranges from 0 to 10, with higher values indicating better image quality. Only scans with an image-quality score ≥ 7 were considered acceptable for analysis. Scans with an image-quality score < 7 were excluded. In addition, scans showing motion artifacts, decentration, incomplete acquisition, or segmentation errors affecting the macular or peripapillary RNFL boundaries were excluded from the analysis. Only the right eye of each participant was included in the statistical analysis. This single-eye approach was applied consistently across all study groups to avoid potential bias arising from inter-eye correlation and to ensure a uniform eye-selection criterion.

### 2.8. Laboratory Parameters and İnflammatory İndices

Laboratory measurements obtained closest to the date of the OCT examination (within ±10 days) were recorded for analysis. Glucose and lipid profile measurements (total cholesterol, HDL-C, LDL-C, and triglycerides) were obtained from fasting blood samples in all participants. The evaluated parameters included complete blood count parameters [white blood cell count (WBC), hemoglobin, platelet count, neutrophil count, lymphocyte count, and monocyte count], fasting glucose, urea, creatinine, aspartate aminotransferase (AST), alanine aminotransferase (ALT), total cholesterol, high-density lipoprotein cholesterol (HDL-C), low-density lipoprotein cholesterol (LDL-C), triglycerides, C-reactive protein (CRP), thyroid function tests [thyroid-stimulating hormone (TSH), free thyroxine (fT4), and free triiodothyronine (fT3)], and thyroid autoimmunity markers, including anti-thyroid peroxidase antibody (anti-TPO) and anti-thyroglobulin antibody (anti-Tg).

Routine biochemical parameters, including glucose, urea, creatinine, liver enzymes, lipid profile, and C-reactive protein (CRP), were measured using an AU5800 automated chemistry analyzer (Beckman Coulter, Brea, CA, USA). Complete blood count parameters were measured using an XN-1000 automated hematology analyzer (Sysmex Corporation, Kobe, Japan).

Thyroid function tests (TSH, fT3, and fT4) and thyroid autoantibodies (anti-TPO and anti-Tg) were measured by chemiluminescent immunoassay using the UniCel DxI 800 Access Immunoassay System (Beckman Coulter, Brea, CA, USA), which was used throughout the study period. The laboratory reference intervals were 0.35–5.10 mIU/L for TSH, 1.8–4.2 pg/mL for fT3, 0.5–1.4 ng/dL for fT4, 0–9 IU/mL for anti-TPO, and 0–4 IU/mL for anti-Tg. CRP was measured in the hospital central laboratory using the routine automated clinical chemistry method, with <5 mg/L considered within the reference range.

Systemic inflammatory indices were calculated from complete blood count parameters as follows: NLR = neutrophil/lymphocyte, PLR = platelet/lymphocyte, MLR = monocyte/lymphocyte, and SII = platelet × neutrophil/lymphocyte [9,10].

These indices were included in the statistical analyses together with thyroid function and autoimmunity parameters.

### 2.9. Outcome Measures

The primary outcome of the study was the comparison of CMT and cpRNFL thickness parameters (temporal, nasal, superior, and inferior quadrants) among the GO+, GO−, and control groups.

Secondary outcomes included the evaluation of the relationships between OCT-derived parameters and systemic inflammatory indices, thyroid function tests, and thyroid autoimmunity markers (anti-TPO and anti-Tg).

### 2.10. Sample Size and Power Consideration

As this was a retrospective study, no a priori sample size calculation was performed, and all eligible participants meeting the predefined inclusion and exclusion criteria during the study period were included. To provide a power justification for the achieved sample size, a sensitivity analysis was performed based on the final sample of 161 participants distributed across three groups (control, *n* = 60; GO+, *n* = 50; GO−, *n* = 51). Assuming a two-sided α level of 0.05 and 80% power, the achieved sample size was sufficient to detect an overall effect size of approximately Cohen’s f = 0.25 in a three-group comparison, corresponding to a moderate effect. Therefore, smaller between-group effects may not have been detected with the available sample size.

### 2.11. Statistical Analysis

Statistical analyses were performed using IBM SPSS Statistics software (version 23.0; IBM Corp., Armonk, NY, USA). Continuous variables were expressed as mean ± standard deviation (SD) for normally distributed data and as median with interquartile range (IQR) when the assumption of normality was not met. Categorical variables were presented as frequencies (*n*) and percentages (%). The normality of data distribution was assessed using the Kolmogorov–Smirnov test.

The three-group OCT comparison (control, GO+, and GO−) was considered the primary comparative analysis. The five OCT outcomes (CMT and superior, inferior, temporal, and nasal cpRNFL thickness) constituted a single primary multiplicity family. For these outcomes, the Kruskal–Wallis test was used for overall three-group comparisons, and the Benjamini–Hochberg procedure was applied across the five omnibus tests to control the false discovery rate (FDR). For OCT outcomes remaining significant after FDR correction (*q* < 0.05), pairwise comparisons were performed using Dunn’s post hoc test with Bonferroni correction. The pooled Graves’-versus-control OCT analysis was considered secondary/exploratory and was retained to provide a complementary overall disease-level comparison. Laboratory comparisons were also considered exploratory and their nominal *p* values were interpreted accordingly. Given their descriptive and hypothesis-generating purpose, no additional multiplicity adjustment was applied across the laboratory variables; therefore, these nominal *p* values were not interpreted as confirmatory evidence. Comparisons between two groups (control vs. pooled Graves’ disease) were performed using the Mann–Whitney U test for continuous variables. Categorical variables were evaluated using the chi-square test or Fisher’s exact test, as appropriate.

Systemic inflammatory indices calculated from complete blood count parameters (NLR, PLR, MLR, and SII) were compared between groups and were also included in correlation analyses. Relationships between OCT parameters (CMT and sectoral cpRNFL measurements) and laboratory variables, including inflammatory indices and thyroid-related parameters, were assessed using Spearman correlation analysis. In selected analyses, partial correlation was additionally performed to adjust for possible confounding effects of age and gender. To account for multiple testing in the correlation analyses, the Benjamini–Hochberg procedure was applied jointly across all 85 correlation tests to control the false discovery rate (FDR). Both unadjusted *p* values and FDR-adjusted *p* values (*q* values) were reported, and *q* < 0.05 was considered statistically significant.

Because raw anti-TPO values were markedly right-skewed (skewness = 2.627), a natural logarithmic transformation [ln(anti-TPO + 1)] was applied, which reduced the skewness to 0.544. Multivariable linear regression was performed among patients with Graves’ disease (*n* = 101) to identify factors associated with inferior cpRNFL thickness. This regression analysis was exploratory and post hoc. Inferior cpRNFL thickness was selected as the dependent variable based on the preceding univariable findings, particularly the observed difference between the GO+ and GO− groups. Ophthalmopathy status was included as the exposure of interest, while age, sex, and log-transformed anti-TPO [ln(anti-TPO + 1)] were included as covariates to assess whether the observed association with ophthalmopathy status persisted after adjustment for these available variables. Model assumptions, multicollinearity, and influential observations were assessed using residual diagnostics, tolerance/VIF values, leverage, and Cook’s distance. Regression coefficients with 95% confidence intervals were reported, with *p* < 0.05 considered statistically significant.

## 3. Results

A total of 161 subjects were included in the analysis, consisting of 60 healthy controls (37.3%), 50 patients in the GO+ group (31.1%), and 51 patients in the GO− group (31.7%). The mean ages were 44.20 ± 7.30 years in the control group, 44.64 ± 10.75 years in the GO+ group, and 45.55 ± 9.35 years in the GO− group. No statistically significant difference in age was observed among the three groups (*p* = 0.735).

The distribution of sex was comparable across the study groups. Female subjects constituted 50.0% (*n* = 30) of the control group, 54.0% (*n* = 27) of the GO+ group, and 51.0% (*n* = 26) of the GO− group, with no statistically significant difference (*p* = 0.912).

As a secondary/exploratory analysis, patients with Graves’ disease (*n* = 101) were pooled and compared with the control group (*n* = 60) to provide a complementary overall disease-level comparison. Nominal between-group differences were observed for superior cpRNFL thickness (121.65 ± 18.06 vs. 127.68 ± 17.96 µm, nominal *p* = 0.042), inferior cpRNFL thickness (123.71 ± 25.87 vs. 136.65 ± 20.01 µm, nominal *p* = 0.001), and temporal cpRNFL thickness (72.10 ± 12.27 vs. 78.32 ± 8.11 µm, nominal *p* < 0.001), with lower values in the pooled Graves’ disease group. No nominal between-group differences were observed for CMT or nasal cpRNFL thickness (both nominal *p* > 0.05) (Table 1, Figure 4).

As part of the exploratory secondary analyses, systemic inflammatory indices calculated from complete blood count parameters (NLR, PLR, MLR, and SII) were compared between the control group and patients with Graves’ disease. No nominal between-group differences were observed for NLR (2.27 ± 2.25 vs. 2.34 ± 1.49, nominal *p* = 0.616), PLR (131.27 ± 63.41 vs. 145.14 ± 87.20, nominal *p* = 0.430), MLR (0.29 ± 0.11 vs. 0.28 ± 0.11, nominal *p* = 0.265), or SII (568.91 ± 474.19 vs. 637.84 ± 422.25, nominal *p* = 0.298) (Table 2).

In the exploratory laboratory analyses, findings for the control and pooled Graves’ groups are summarized in Table 3. CRP levels were higher in patients with Graves’ disease compared with the control group (3.87 ± 3.40 vs. 1.86 ± 1.11 mg/L, nominal *p* < 0.001). Urea values were also higher in the Graves’ group (28.88 ± 9.80 vs. 23.91 ± 7.19 mg/dL, nominal *p* = 0.001).

No nominal between-group differences were observed for WBC, hemoglobin, platelet count, neutrophil, lymphocyte, monocyte, glucose, AST, ALT, creatinine, total cholesterol, HDL, LDL, or triglyceride levels (all nominal *p* > 0.05).

In these exploratory comparisons, several thyroid-related laboratory parameters showed nominal between-group differences. TSH levels were lower in the Graves’ group than in controls (0.97 ± 1.21 vs. 1.78 ± 0.80 mIU/L, nominal *p* < 0.001), whereas fT3 (3.94 ± 1.87 vs. 3.26 ± 0.42 pg/mL, nominal *p* = 0.006) and fT4 (1.14 ± 0.63 vs. 0.85 ± 0.11 ng/dL, nominal *p* = 0.001) levels were higher in patients with Graves’ disease. Anti-TPO concentrations were higher in the Graves’ group compared with controls (118.22 ± 245.34 vs. 0.77 ± 0.31 IU/mL, nominal *p* < 0.001) (Table 3).

In the primary three-group OCT analysis, significant overall differences were observed for superior cpRNFL (*p* = 0.010, FDR-adjusted *q* = 0.0125), inferior cpRNFL (*p* < 0.001, *q* = 0.0024), temporal cpRNFL (*p* = 0.002, *q* = 0.0050), and nasal cpRNFL (*p* = 0.006, *q* = 0.0092). All four findings remained statistically significant after Benjamini–Hochberg FDR correction across the five primary OCT outcomes. In contrast, CMT did not differ significantly among the three groups (*p* = 0.925, *q* = 0.925). Superior cpRNFL thickness was lower in the GO− group than in the control group (117.67 ± 17.15 vs. 127.68 ± 17.96 µm, post hoc *p* = 0.010). No significant difference was observed between the GO+ and control groups or between the two Graves’ subgroups (Table 4).

Inferior cpRNFL thickness showed a stepwise reduction, with the lowest values in the GO− group. The GO− group had significantly lower inferior cpRNFL thickness than both the control group (116.39 ± 27.52 vs. 136.65 ± 20.01 µm, post hoc *p* < 0.001) and the GO+ group (116.39 ± 27.52 vs. 131.18 ± 21.90 µm, post hoc *p* = 0.005).

Temporal cpRNFL thickness was reduced in both Graves’ subgroups compared with controls. The GO+ group (70.94 ± 12.15 µm) and the GO− group (73.24 ± 12.40 µm) had significantly lower values than the control group (78.32 ± 8.11 µm; post hoc *p* = 0.002 and post hoc *p* = 0.041, respectively), whereas no difference was detected between the GO+ and GO− groups (Table 4).

Nasal cpRNFL thickness was significantly lower in the GO− group compared with both the control group (77.71 ± 15.33 vs. 85.53 ± 11.39 µm, post hoc *p* = 0.008) and the GO+ group (77.71 ± 15.33 vs. 84.76 ± 14.30 µm, post hoc *p* = 0.028) (Table 4, Figure 5).

In the exploratory three-group analyses of systemic inflammatory indices, an overall difference was observed only for MLR (nominal *p* = 0.034). The highest mean MLR value was observed in the GO− group (0.32 ± 0.12), whereas lower values were found in the control group (0.28 ± 0.11) and the GO+ group (0.27 ± 0.09) (Figure 6). However, post hoc pairwise analyses did not reveal statistically significant differences between individual groups after Bonferroni correction.

No nominal overall differences were observed among the three groups for NLR, PLR, or SII (nominal *p* = 0.795, *p* = 0.476, and *p* = 0.570, respectively) (Table 5).

In the exploratory three-group laboratory analyses, several thyroid-related parameters showed overall differences at the nominal significance level. In the direct comparison between the two Graves’ disease subgroups, fT3 levels were higher in the GO+ group than in the GO− group (4.34 ± 2.42 vs. 3.55 ± 0.97 pg/mL, post hoc *p* = 0.023), whereas TSH, fT4, anti-Tg, and anti-TPO levels did not differ between the GO+ and GO− groups. Compared with controls, both Graves’ disease subgroups had lower TSH levels, while fT4 was higher in the GO+ group. Anti-Tg and anti-TPO levels were also higher in the GO+ group than in controls. Although anti-Tg levels did not differ between the control and combined Graves’ disease groups in the exploratory two-group analysis (nominal *p* = 0.079), an overall difference was observed in the exploratory three-group analysis (nominal *p* = 0.017), with post hoc analysis showing higher anti-Tg levels in the GO+ group than in controls. This pattern indicates that the three-group difference in anti-Tg was mainly attributable to the GO+ subgroup. Detailed overall and post hoc comparisons are presented in Table 6.

Among the biochemical and hematological variables examined in these exploratory analyses, glucose, urea, HDL, and CRP showed overall differences at the nominal significance level. Glucose levels were higher in the GO− group than in controls (97.65 ± 10.87 vs. 91.52 ± 10.42 mg/dL, post hoc *p* = 0.016). Urea values were also higher in the GO− group than in controls (29.94 ± 9.01 vs. 23.91 ± 7.19 mg/dL, post hoc *p* = 0.001). HDL levels were higher in the GO− group than in the GO+ group (53.75 ± 11.81 vs. 48.64 ± 9.49 mg/dL, post hoc *p* = 0.037), whereas no post hoc difference was observed between the GO− group and controls (Table 6). CRP levels were higher in both the GO+ group (3.22 ± 3.14 mg/L) and the GO− group (4.51 ± 3.56 mg/L) than in the control group (1.86 ± 1.11 mg/L; post hoc *p* = 0.028 and post hoc *p* < 0.001, respectively), whereas no post hoc difference was observed between the two Graves’ subgroups (Figure 7).

No nominal overall differences were observed among the groups for WBC, hemoglobin, platelet count, neutrophil, lymphocyte, monocyte, AST, ALT, creatinine, total cholesterol, LDL, or triglyceride levels (all nominal *p* > 0.05) (Table 6).

Spearman correlation analysis identified several weak associations that reached nominal statistical significance before correction for multiple testing. Superior cpRNFL thickness was negatively correlated with fT4 levels (*ρ* = −0.174, raw *p* = 0.027), inferior cpRNFL thickness showed a weak negative correlation with CRP levels (*ρ* = −0.157, raw p = 0.047) and age (*ρ* = −0.174, raw *p* = 0.028), and temporal cpRNFL thickness showed a weak negative correlation with anti-TPO levels (*ρ* = −0.163, raw *p* = 0.038). However, after Benjamini–Hochberg false discovery rate correction across the 85 correlation tests reported in Table 7 and Table 8, none of these associations remained statistically significant (all FDR-adjusted *p* [*q*] = 0.5153). The association between CRP and inferior cpRNFL thickness was also not significant after adjustment for age and gender in partial correlation analysis (*r* = −0.092, *p* = 0.247).

Among the systemic inflammatory indices, NLR showed a weak positive correlation with nasal cpRNFL thickness that reached nominal significance before multiple-testing correction (*ρ* = 0.156, raw *p* = 0.049). However, this association did not remain statistically significant after Benjamini–Hochberg FDR correction (*q* = 0.5153). It was also not significant after adjustment for age and gender in partial correlation analysis (*r* = 0.110, *p* = 0.169). No correlations between PLR, MLR, or SII and the OCT parameters reached nominal statistical significance, and none of the correlations involving systemic inflammatory indices remained significant after FDR correction (Table 8).

In the multivariable analysis, the regression was restricted to patients with Graves’ disease (*n* = 101), with inferior cpRNFL thickness as the dependent variable. Ophthalmopathy status, age, sex, and ln (anti-TPO + 1) were included as predictors. The overall model was statistically significant (F(4,96) = 3.294, *p* = 0.014), explaining 12.1% of the variance in inferior cpRNFL thickness (R² = 0.121; adjusted R² = 0.084). GO+ status remained associated with greater inferior cpRNFL thickness compared with GO− status after adjustment for age, sex, and log-transformed anti-TPO (B = 12.926 µm, 95% CI: 2.831 to 23.020; *β* = 0.251; *p* = 0.013). In contrast, age (*p* = 0.184), sex (*p* = 0.955), and log-transformed anti-TPO (*p* = 0.238) were not significantly associated with inferior cpRNFL thickness in this model. Multicollinearity was not evident, with tolerance values ranging from 0.842 to 0.940 and VIF values ranging from 1.064 to 1.188. Influence diagnostics did not indicate highly influential observations (maximum Cook’s distance = 0.096); standardized residuals ranged from −2.862 to 2.318 (Table 9).

## 4. Discussion

In the present study, we evaluated the association between systemic inflammatory indices, thyroid autoimmunity markers, and optical coherence tomography–derived neuroretinal measurements in patients with Graves’ disease, with a particular focus on differences according to ophthalmopathy status. The main findings of this study can be summarized as follows: (1) in the primary three-group analysis, significant differences were observed in superior, inferior, temporal, and nasal cpRNFL thickness among the control, GO+, and GO− groups, whereas CMT did not differ among the groups; (2) cpRNFL thinning was more pronounced in the GO− subgroup than in the GO+ subgroup for several quadrants; (3) systemic inflammatory indices derived from complete blood count parameters were largely similar between groups, while exploratory laboratory analyses showed higher CRP levels in Graves’ disease at the nominal significance level; and (4) selected OCT parameters showed weak correlations with thyroid hormone levels, CRP, and anti-TPO that reached nominal significance but did not remain statistically significant after FDR correction, while GO+ status remained associated with greater inferior cpRNFL thickness compared with GO− status after adjustment for age, sex, and log-transformed anti-TPO in the exploratory multivariable analysis restricted to patients with Graves’ disease. Recent literature increasingly supports the concept that thyroid eye disease represents a heterogeneous immunoinflammatory spectrum in which structural ocular changes, biochemical activity, and clinical severity do not always progress in parallel [11,12].

In the secondary/exploratory pooled analysis, superior, inferior, and temporal cpRNFL thickness values were lower in the overall Graves’ cohort than in controls at the nominal significance level, whereas CMT did not differ between the groups. This pattern is compatible with previous OCT-based studies showing that the cpRNFL is particularly vulnerable to autoimmune, inflammatory, and microvascular disturbances in thyroid-related disease. Recent systematic reviews have emphasized that cpRNFL and ganglion cell complex measurements may reveal early structural changes even in patients without dysthyroid optic neuropathy, suggesting that subclinical neuroaxonal damage may develop before overt visual impairment becomes apparent [12,13]. Because the cpRNFL reflects the integrity of retinal ganglion cell axons, thinning in this layer may indicate chronic neurodegenerative or ischemic stress rather than acute inflammatory swelling. Therefore, the selective involvement of cpRNFL rather than macular thickness observed in our cohort is biologically plausible and consistent with the current understanding of thyroid-related ocular pathology [14]. 

However, cpRNFL thinning in Graves’ ophthalmopathy should not be attributed exclusively to inflammatory or autoimmune mechanisms. Orbital tissue expansion, including enlargement of the extraocular muscles and increased orbital soft-tissue volume, may increase orbital pressure and contribute to mechanical compression of the optic nerve and subsequent axonal injury. Therefore, the observed cpRNFL changes may reflect the combined effects of inflammatory, vascular, metabolic, and mechanical factors rather than a single pathogenic mechanism. Furthermore, the effect of compressive optic nerve involvement on cpRNFL thickness may vary according to disease stage and activity. Early or active compressive changes may be associated with axonal swelling and increased RNFL thickness, whereas chronic injury may eventually result in axonal loss and RNFL thinning. Because standardized GO activity and severity staging was unavailable in our retrospective cohort, the GO+ group may have included patients at different pathophysiological stages. Therefore, relatively preserved cpRNFL thickness in the GO+ group cannot be assumed to indicate absence of structural involvement and may, at least in part, reflect stage-dependent differences in RNFL response.

An unexpected finding in our study was that cpRNFL thinning appeared more pronounced in the GO− subgroup than in the GO+ subgroup for several sectors. This observation should be interpreted cautiously. Rather than indicating that clinically unapparent disease is necessarily more severe, several alternative explanations should be considered: (1) heterogeneity in disease phenotype and inflammatory activity; (2) differences in disease duration; (3) differences in treatment exposure; and (4) residual confounding inherent to the retrospective study design.

Previous studies have suggested that structural and microvascular alterations may occur even in the absence of clinically overt ophthalmopathy, supporting the concept that thyroid eye disease represents a heterogeneous spectrum rather than a strictly binary condition [13,15,16].

Importantly, GO classification in the present study was based on retrospectively documented clinical findings, while standardized activity and severity assessments, uniform exophthalmometric thresholds, and orbital imaging were not available for all patients. Therefore, some patients with mild or subclinical ophthalmopathy may have been classified as GO−, and misclassification cannot be excluded. Accordingly, the greater cpRNFL thinning observed in the GO− subgroup should be regarded as hypothesis-generating rather than definitive and should not be interpreted as evidence that the absence of clinically apparent ophthalmopathy is associated with greater neuroretinal damage. Unmeasured factors such as disease duration, treatment status, smoking, and disease activity may also have influenced the structural findings. Prospective studies incorporating standardized activity and severity grading, objective exophthalmometric measurements, orbital imaging, and longitudinal follow-up are required to determine whether neuroretinal alterations occur independently of clinically overt orbital involvement and to clarify the clinical significance of these findings.

The finding that temporal cpRNFL thickness was reduced in both GO+ and GO− groups compared with controls is also noteworthy. The temporal quadrant corresponds largely to the papillomacular bundle, which is known to be highly sensitive to metabolic stress, ischemia, and early optic nerve dysfunction. Previous OCT studies in thyroid-associated ophthalmopathy have reported that temporal and inferior RNFL sectors may be affected earlier than other quadrants, possibly because of their higher metabolic demand and susceptibility to perfusion abnormalities. Even in the absence of clinically diagnosed dysthyroid optic neuropathy, subtle temporal cpRNFL thinning may indicate early optic nerve involvement. Recent clinical imaging studies have highlighted the value of OCT in detecting structural alterations before the development of visual symptoms, supporting the clinical relevance of quadrant-specific RNFL changes in autoimmune thyroid disease [17,18]. 

In contrast to the cpRNFL findings, macular thickness did not differ significantly among the control, GO+, and GO− groups. This result is consistent with several recent reports indicating that macular thickness may remain normal in patients without dysthyroid optic neuropathy, whereas cpRNFL or ganglion cell layer parameters are more sensitive indicators of early damage. The macular region has a different vascular supply and structural organization compared with the peripapillary region and may be less affected by mild perfusion disturbances or low-grade inflammation. Therefore, the absence of macular changes in our study does not exclude retinal involvement but rather suggests that cpRNFL measurements may be more useful for detecting subclinical neuroretinal alterations in Graves’ disease [14,19]. 

When systemic inflammatory markers were evaluated, we found that NLR, PLR, and SII were not significantly different between patients with Graves’ disease and controls, and only MLR showed a modest difference in the three-group comparison that did not remain significant after post hoc analysis. These findings differ from some recent studies reporting elevated CBC-derived inflammatory indices in patients with active or moderate-to-severe thyroid eye disease. However, previous investigations often focused on patients with clinically active orbitopathy, higher disease severity, or abnormal thyroid function, whereas our cohort included both GO+ and GO− patients regardless of activity status. Recent meta-analyses have emphasized that blood count–derived inflammatory ratios are more strongly associated with disease activity than with the mere presence of Graves’ disease, which may explain the relatively weak differences observed in our study [20]. 

In contrast to the CBC-derived indices, exploratory analyses showed higher CRP levels in patients with Graves’ disease than in controls, with higher levels also observed in both the GO+ and GO− subgroups. Given the exploratory nature of these laboratory comparisons and the absence of multiplicity adjustment across laboratory variables, this finding should be interpreted as hypothesis-generating rather than confirmatory evidence of systemic inflammatory activation in Graves’ disease. CRP reflects hepatic acute-phase response and may capture broader inflammatory activity than leukocyte-based indices, which are influenced by individual variability in blood cell distribution. A weak negative correlation between CRP and inferior cpRNFL thickness reached nominal significance; however, this association did not remain statistically significant after FDR correction and was also not significant after adjustment for age and sex. Therefore, this finding should not be interpreted as evidence of an independent association between systemic inflammation and cpRNFL thickness. Recent studies have similarly reported that systemic inflammatory markers may show modest but inconsistent associations with structural ocular parameters in thyroid eye disease, indicating that inflammation alone does not fully explain retinal changes [21]. In our analysis, the weak nominal association between CRP and cpRNFL thickness did not remain statistically significant after FDR correction or adjustment for age and sex, further supporting the need for cautious interpretation.

Exploratory analyses also identified nominal differences in several thyroid-related biochemical parameters between the study groups. TSH levels were lower and fT3 and fT4 levels were higher in patients with Graves’ disease, particularly in the GO+ subgroup; however, these laboratory comparisons were exploratory and were not adjusted for multiplicity across laboratory variables. Excess thyroid hormone may affect ocular tissues through oxidative stress, endothelial dysfunction, altered blood flow, and impaired microvascular regulation, potentially increasing susceptibility to neural injury [22,23]. In our study, thyroid hormone levels showed weak nominal associations with selected cpRNFL parameters; however, these associations did not remain statistically significant after FDR correction. Therefore, their potential relationship with neuroretinal structure should be considered hypothesis-generating and requires confirmation in future studies.

The role of thyroid autoimmunity was also evaluated in our study. In the exploratory laboratory analysis, anti-TPO levels were higher in the Graves’ group than in controls at the nominal significance level; however, their weak negative correlation with temporal cpRNFL thickness did not remain statistically significant after FDR correction and should therefore be interpreted cautiously. Recent biomarker studies have shown that thyroid eye disease is associated with complex immune dysregulation involving cytokines, fibroblast activation, oxidative stress pathways, and immune-checkpoint signaling rather than a single antibody-mediated mechanism [24,25]. Further prospective studies with larger cohorts are needed to clarify whether thyroid autoimmunity is independently associated with neuroretinal structural alterations.

In the exploratory post hoc multivariable regression analysis restricted to patients with Graves’ disease, GO+ status remained associated with greater inferior cpRNFL thickness compared with GO− status after adjustment for age, sex, and log-transformed anti-TPO. However, this finding should be interpreted cautiously given the limited explanatory capacity of the model (adjusted R² = 0.084) and the possibility of residual confounding by clinical variables not available in this retrospective dataset. In particular, disease duration, smoking status, treatment exposure, standardized thyroid disease activity, GO activity and severity, and axial length were not consistently available and therefore could not be incorporated into the model. Accordingly, this association should not be interpreted as indicating a protective effect of ophthalmopathy or greater disease severity in the GO− group. Rather, it may reflect heterogeneity in disease phenotype, activity, treatment exposure, ocular biometry, or other unmeasured factors. Similar complexity in the relationship between immunological markers and clinical manifestations has been noted in recent clinical and guideline-based literature on thyroid eye disease [26]. Therefore, these regression findings should be considered exploratory rather than confirmatory and require validation in prospective studies incorporating standardized assessments of ophthalmopathy activity and severity.

Another noteworthy observation was the lack of strong correlations between OCT parameters and CBC-derived inflammatory indices. Although some studies have reported elevated NLR, PLR, or SII in active thyroid eye disease, these markers appear to be more closely related to inflammatory activity than to structural damage. Recent reviews have emphasized that hematologic indices are useful for evaluating systemic inflammation but have limited specificity for predicting ocular structural changes [21,27]. Although some weak nominal associations were observed for CRP and thyroid autoimmunity markers in our results, none of the evaluated biomarker–OCT correlations remained statistically significant after FDR correction. These findings indicate that the present correlation analyses do not provide robust evidence for an association between the evaluated systemic biomarkers and neuroretinal structural alterations.

From a clinical perspective, OCT may provide complementary information on neuroretinal structure in selected patients with Graves’ disease. However, given the retrospective cross-sectional design and lack of standardized activity and severity assessment, the clinical significance of these findings requires confirmation in prospective longitudinal studies [27,28].

The present study has several strengths that should be considered alongside its limitations. First, it evaluated OCT measurements, systemic inflammatory indices, thyroid hormones, and thyroid autoimmunity parameters within the same cohort, allowing a comprehensive analysis of potential interactions. Second, the inclusion of both GO+ and GO− subgroups together with healthy controls enabled us to examine whether neuroretinal changes depend on ophthalmopathy status. Third, all OCT measurements were obtained using the same device and protocol, reducing measurement variability. These features provide a more integrated view of ocular involvement in Graves’ disease compared with studies focusing on a single parameter.

Although predefined ocular and systemic eligibility criteria were applied, the retrospective identification and selection of participants from individuals attending a single ophthalmology outpatient clinic may have introduced selection bias. Diabetes mellitus, hypertension, chronic kidney disease, and neurologic diseases potentially affecting the RNFL were excluded to reduce potential confounding of OCT measurements; nevertheless, residual confounding from other unmeasured systemic conditions cannot be completely excluded.

However, several limitations should be considered when interpreting the results of this study. The retrospective and cross-sectional design limits the ability to establish causal relationships, and the observed associations cannot be interpreted as evidence of disease progression. Because the analysis relied on routinely recorded clinical data, standardized evaluation of disease activity, duration of Graves’ disease, smoking status, and treatment history was not available for all participants, all of which may influence both inflammatory parameters and OCT measurements. Accordingly, ophthalmopathy status was classified based on clinical documentation in medical records rather than a fully standardized activity- or severity-based assessment. Furthermore, objective exophthalmometric thresholds and orbital imaging were not systematically available and therefore could not be incorporated into a standardized GO classification algorithm. Because some of the clinical findings used to identify ophthalmopathy may be nonspecific, misclassification between the GO+ and GO− groups, particularly in patients with mild or subclinical ophthalmopathy, cannot be excluded. This limitation is especially relevant when interpreting the greater cpRNFL thinning observed in the GO− subgroup. 

Attrition was unequal across the three candidate groups, with exclusion rates of 45.7% (43/94) in the GO− group, 29.6% (21/71) in the GO+ group, and 25.0% (20/80) among controls. Because group-specific reasons for exclusion could not be reliably reconstructed retrospectively, differential selection bias cannot be excluded. Importantly, this unequal attrition, together with potential misclassification arising from the retrospective assessment of ophthalmopathy status, may have influenced the observed between-group differences in cpRNFL thickness, particularly the unexpectedly lower measurements in the GO− subgroup. Therefore, these findings should be regarded as exploratory and hypothesis-generating rather than as definitive evidence of greater neuroretinal involvement in patients without clinically documented ophthalmopathy.

In addition, because patients with clinically diagnosed dysthyroid optic neuropathy (DON) were excluded, the GO+ group may underrepresent the most severe end of the ophthalmopathy spectrum. This selection may have attenuated between-group differences and should be considered when interpreting the structural OCT findings.

Potentially relevant confounding factors such as prior antithyroid therapy, corticosteroid use, orbital disease activity, and metabolic status could not be evaluated systematically. TRAb measurements were not available in all patients, which limited the ability to assess the relationship between disease-specific autoimmunity and neuroretinal changes. In addition, axial length measurements and complete quantitative IOP data were not consistently available because of the retrospective design and therefore could not be incorporated as covariates in the multivariable analyses, although only eyes with IOP within the normal range and without significant refractive error (spherical equivalent > +3.0 D or <−3.0 D) were included. Therefore, the observed OCT differences may partly reflect unmeasured disease phenotype, treatment exposure, ocular biometry, or residual confounding rather than Graves’ disease itself in isolation.

Standardized measures of GO activity and severity, including the Clinical Activity Score (CAS) and EUGOGO severity categories, were not consistently available for all patients because of the retrospective design. Consequently, the GO+ group could not be stratified according to disease activity or severity and should be interpreted as a clinically heterogeneous group defined by the presence of documented ophthalmopathy rather than as a uniform disease stage. This limitation is particularly relevant to the interpretation of cpRNFL findings, because orbital tissue expansion, extraocular muscle enlargement, and compressive effects on the optic nerve may vary substantially according to GO severity. Moreover, without standardized activity and severity staging, we could not distinguish potentially stage-dependent RNFL changes, including early swelling from later axonal thinning. Furthermore, patients with clinically diagnosed dysthyroid optic neuropathy were excluded; therefore, the present findings should not be extrapolated to sight-threatening GO. Accordingly, this study cannot determine whether cpRNFL alterations vary according to GO activity or severity, and prospective studies incorporating standardized CAS and EUGOGO severity grading are required to address this question.

Axial length measurements were not routinely available because of the retrospective study design. Although participants with significant refractive error (spherical equivalent > +3.0 D or <−3.0 D) were excluded, the absence of axial length data remains an important limitation and a potential source of residual measurement variability in cpRNFL comparisons. Furthermore, ganglion cell complex (GCC) analysis, OCT angiography, and orbital imaging were not available. In particular, the absence of GCC measurements limits a more comprehensive assessment of retinal neurodegeneration and prevents attribution of the observed cpRNFL thinning to a specific pathogenic mechanism. Orbital imaging data were also unavailable; therefore, the potential contribution of extraocular muscle enlargement, increased orbital tissue volume, and compressive axonal injury could not be directly evaluated. To address multiplicity, the Benjamini–Hochberg FDR procedure was applied separately to the five primary OCT omnibus tests and to the family of 85 biomarker–OCT correlation tests. Laboratory comparisons were considered exploratory and were interpreted using nominal *p* values without additional across-variable multiplicity adjustment. None of the nominally significant biomarker–OCT correlations remained statistically significant after FDR correction; therefore, these associations should be regarded as exploratory and interpreted with caution.

Finally, the relatively moderate sample size and the single-center design may limit the generalizability of the findings. Prospective, longitudinal studies including standardized activity scoring, detailed antibody profiling, and comprehensive ocular imaging are required to confirm the present observations and to clarify their clinical significance.

## 5. Conclusions

In the primary three-group analysis, significant differences in sectoral cpRNFL thickness were observed among the control, GO+, and GO− groups, whereas CMT did not differ among the groups. Structural differences were also observed in patients classified as GO− based on available clinical records. However, the retrospective classification of ophthalmopathy, potential misclassification of patients with mild or subclinical disease, and the disproportionately higher exclusion rate in the GO− group introduce important concerns regarding selection bias and the validity of between-group comparisons. These limitations, together with potential residual confounding, preclude definitive conclusions regarding the relationship between ophthalmopathy status and neuroretinal structural alterations. CBC-derived inflammatory indices showed limited relationships with OCT parameters. Although several weak correlations involving CRP, thyroid hormone levels, anti-TPO, and NLR reached nominal statistical significance, none remained statistically significant after FDR correction.

Accordingly, the present findings should be considered strictly exploratory and hypothesis-generating rather than evidence of neuroretinal involvement independent of clinically apparent ophthalmopathy or disease progression. Larger prospective longitudinal studies incorporating standardized GO activity and severity assessments, detailed antibody profiling, objective exophthalmometric measurements, orbital imaging, and comprehensive ocular imaging are required to validate these observations and clarify their clinical significance in Graves’ disease.

## Figures and Tables

**Figure 1:**
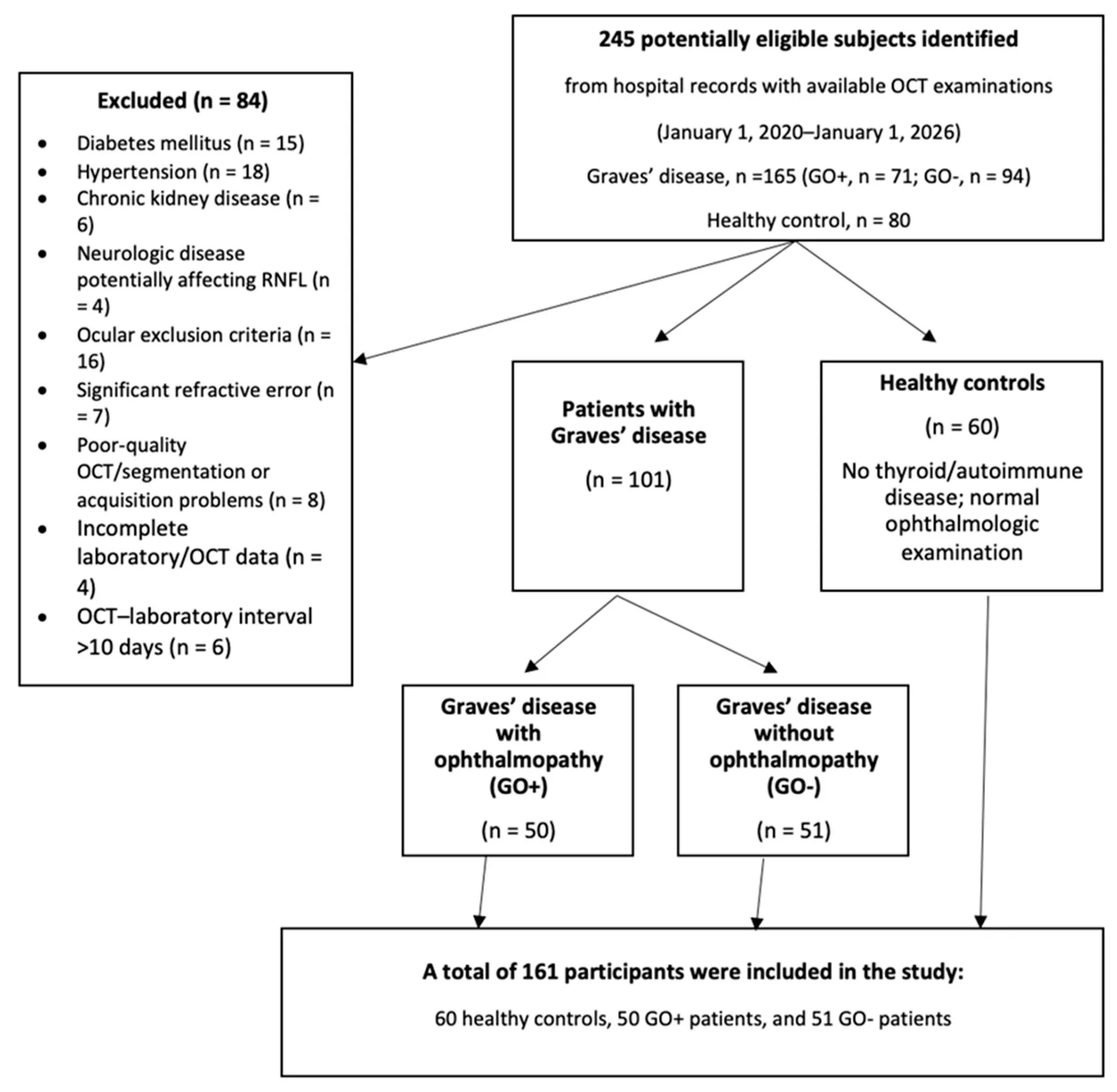
Flow diagram of the study.

**Figure 2:**
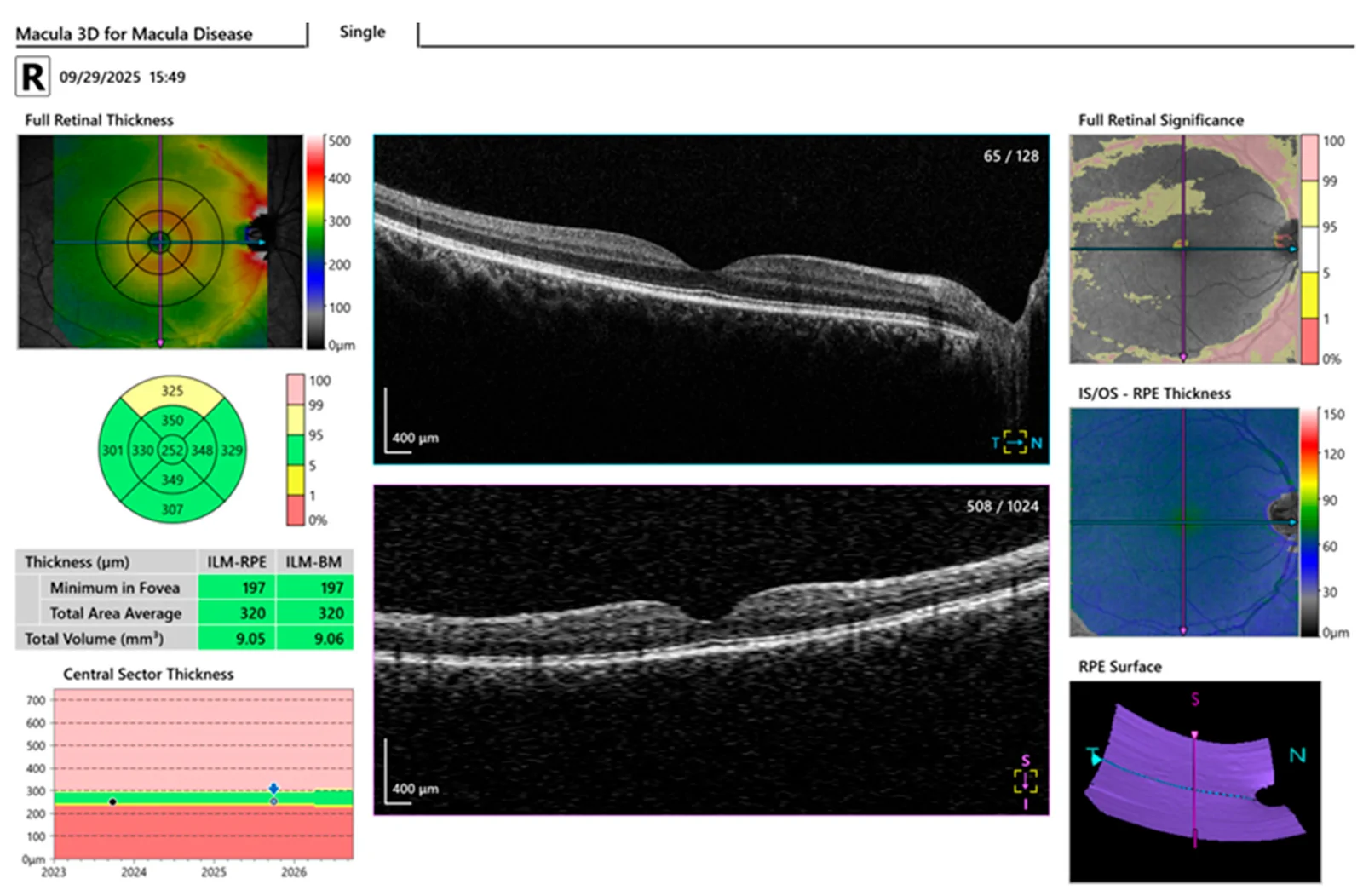
Central macular thickness (CMT) measurement obtained by spectral-domain optical coherence tomography (OCT). CMT represents the retinal thickness measured in the central macular region centered on the fovea.

**Figure 3:**
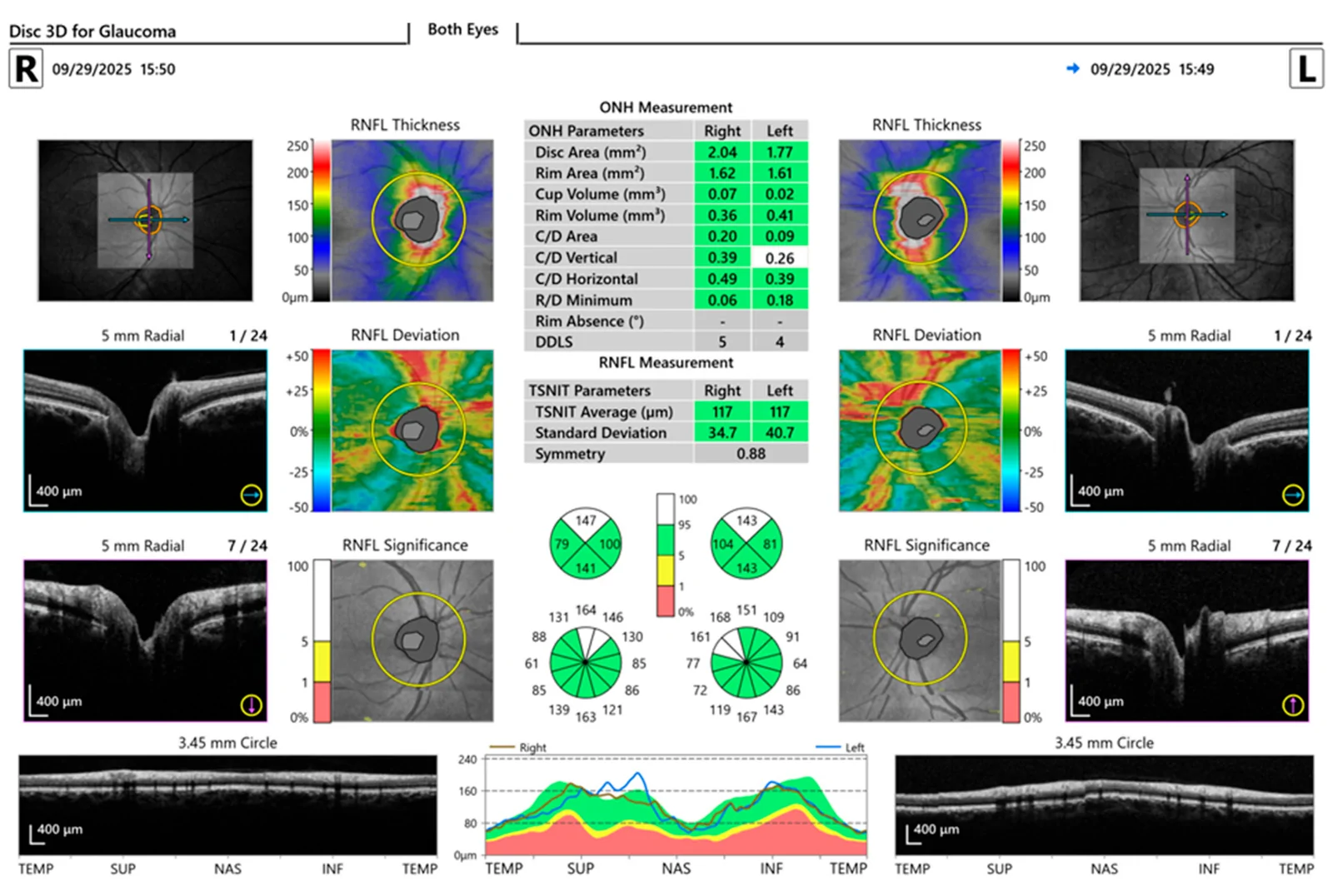
Circumpapillary retinal nerve fiber layer (cpRNFL) measurement obtained by spectral-domain optical coherence tomography (OCT). The peripapillary scan is centered on the optic disc, and cpRNFL thickness is evaluated separately in the superior, inferior, temporal, and nasal quadrants.

**Figure 4:**
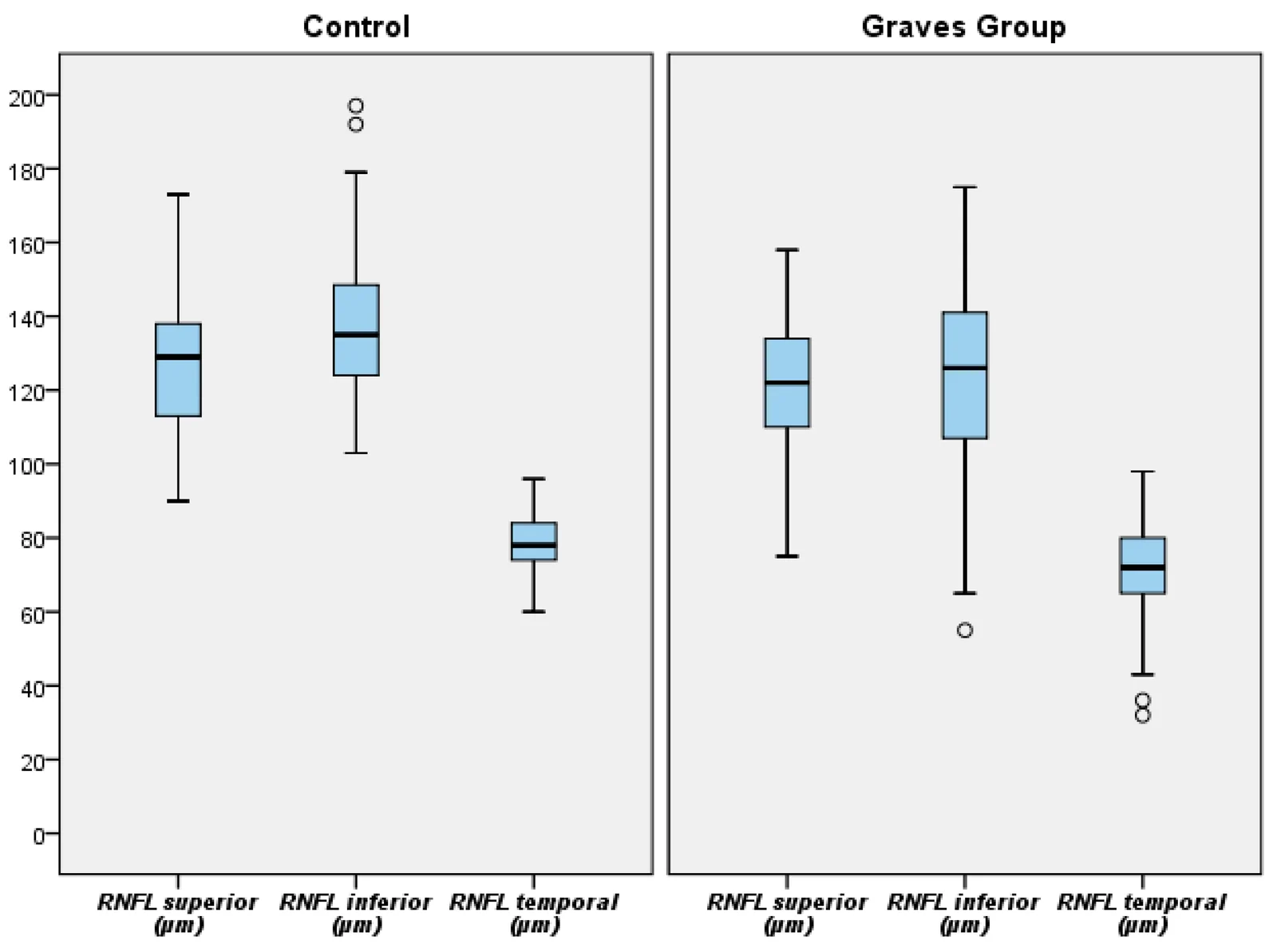
Boxplot distribution of circumpapillary retinal nerve fiber layer (cpRNFL) thickness measurements in the superior, inferior, and temporal quadrants in the control and Graves’ groups. Boxes represent the interquartile range, horizontal lines indicate the median values, and whiskers represent the most extreme non-outlier values. Open circles (○) indicate outliers.

**Figure 5:**
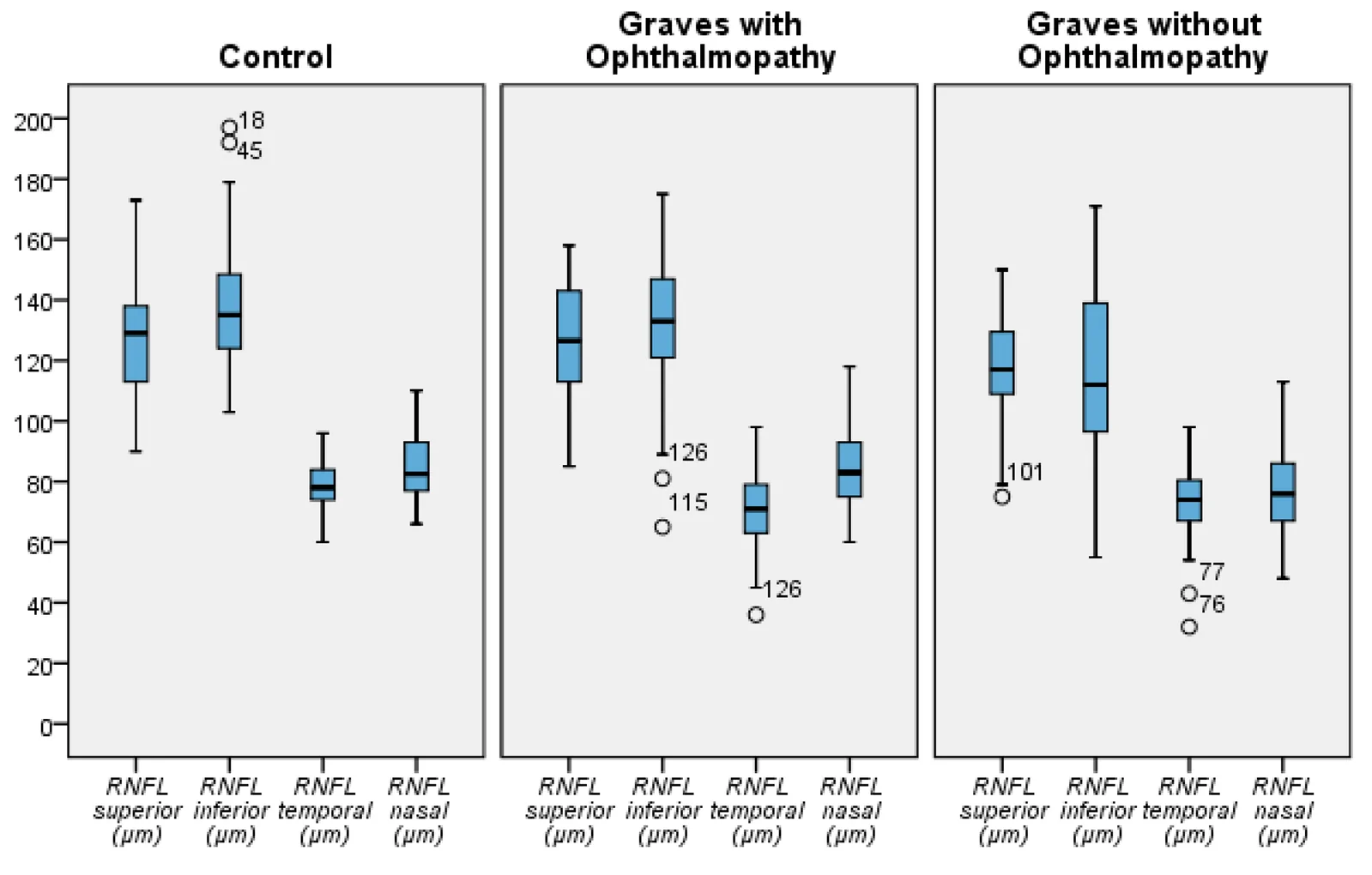
Distribution of circumpapillary retinal nerve fiber layer (cpRNFL) thickness values in the control, GO+, and GO− groups. Box-and-whisker plots show superior, inferior, temporal, and nasal cpRNFL thickness measurements obtained by optical coherence tomography (OCT) in healthy controls, Graves’ disease patients with ophthalmopathy (GO+), and Graves’ disease patients without ophthalmopathy (GO−). Boxes represent the interquartile range (IQR), horizontal lines indicate the median values, and whiskers show the minimum and maximum values excluding outliers. Circles indicate outlier observations. Open circles (○) indicate outlier observations, and the adjacent numbers represent the corresponding case identifiers.

**Figure 6:**
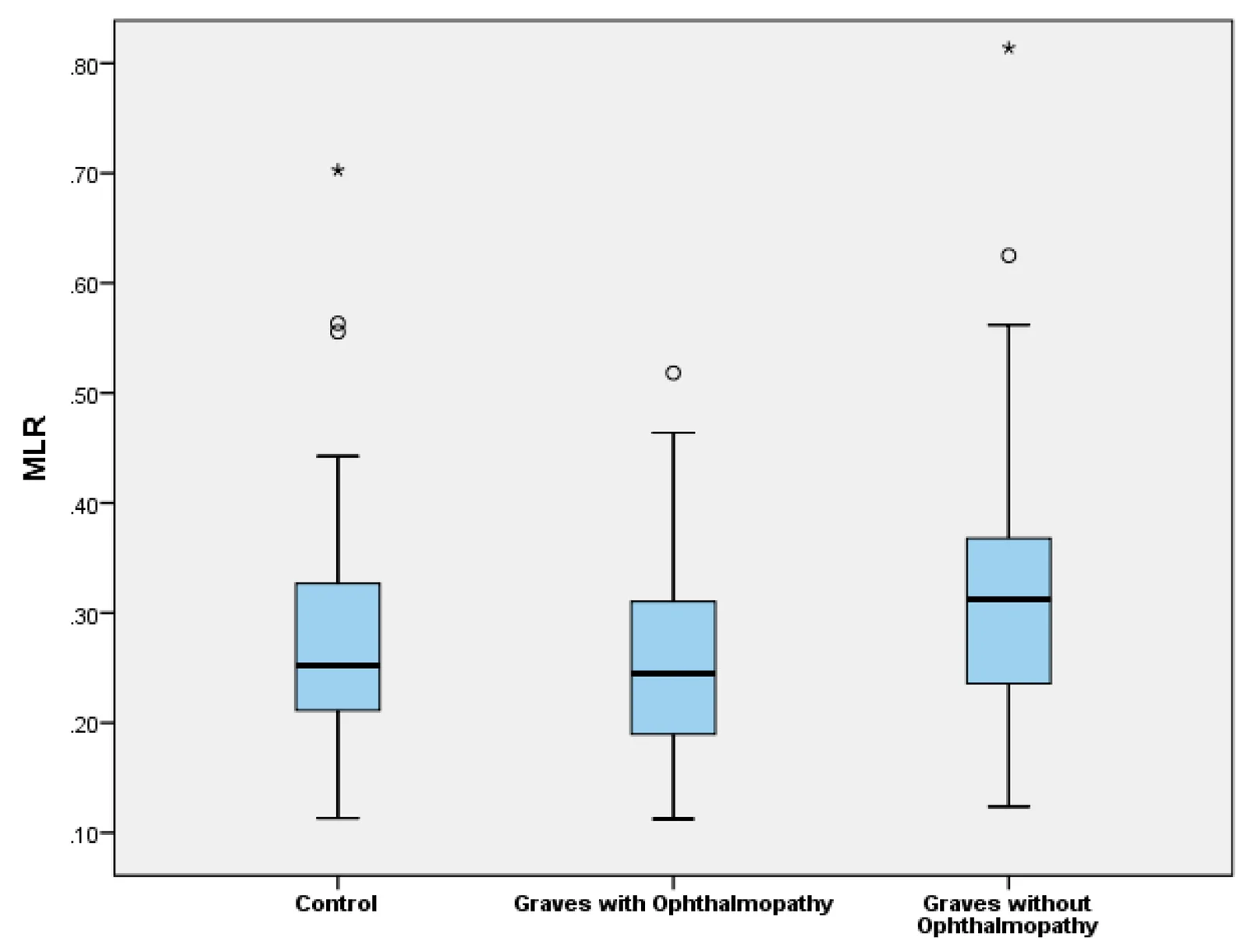
Comparison of monocyte-to-lymphocyte ratio (MLR) among the control, GO+, and GO− groups. Box-and-whisker plots show the distribution of MLR values in healthy controls, Graves’ disease patients with ophthalmopathy (GO+), and Graves’ disease patients without ophthalmopathy (GO−). Boxes represent the interquartile range (IQR), horizontal lines indicate median values, and whiskers show the minimum and maximum values excluding outliers. Circles and asterisks indicate outlier observations.

**Figure 7:**
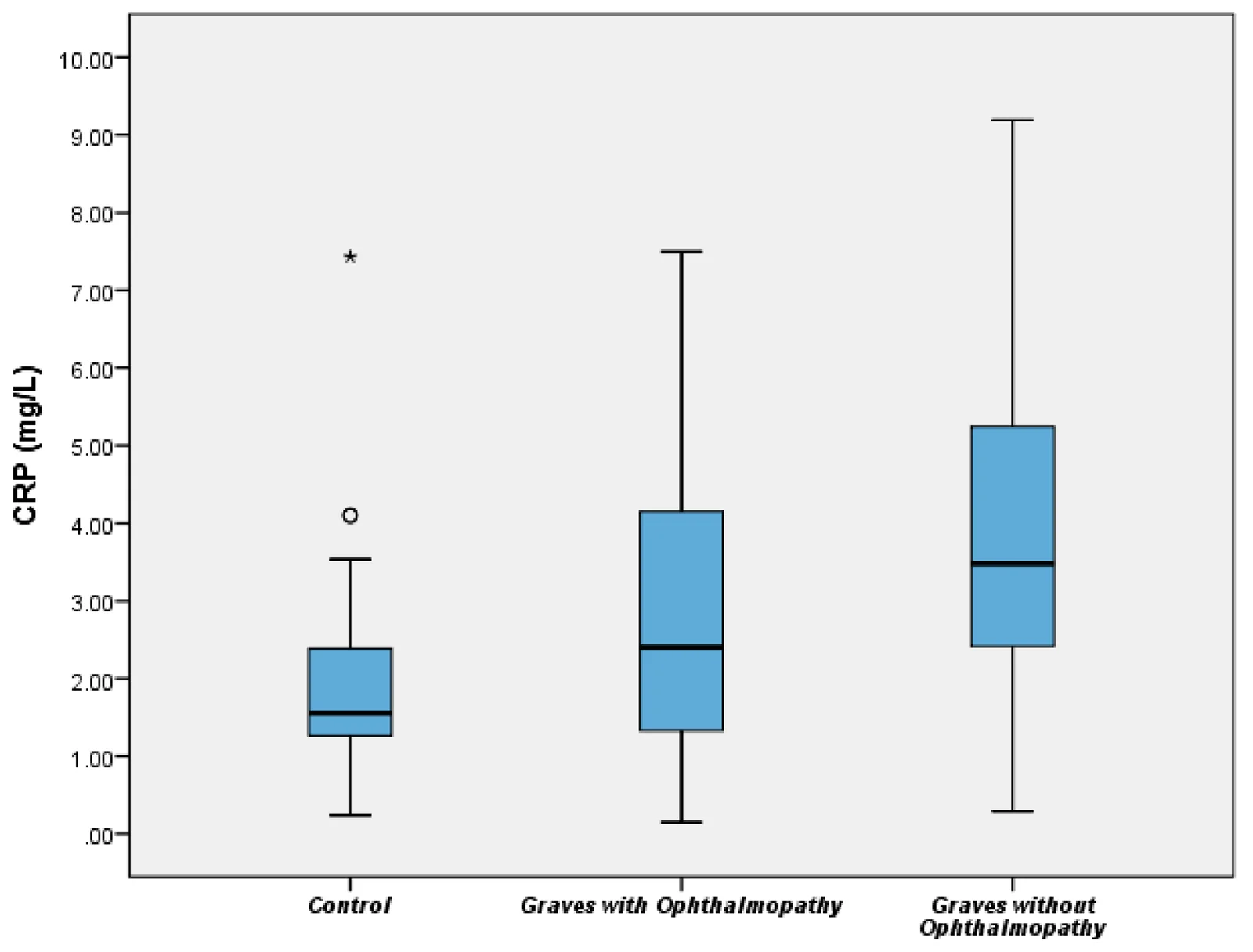
Distribution of C-reactive protein (CRP) levels among the control, GO+, and GO− groups. Box-and-whisker plots show the distribution of serum CRP concentrations (mg/L) in healthy controls, Graves’ disease patients with ophthalmopathy (GO+), and Graves’ disease patients without ophthalmopathy (GO−). Boxes represent the interquartile range (IQR), horizontal lines indicate median values, and whiskers show the minimum and maximum values excluding outliers. Open circles (○) indicate outliers, whereas asterisks (*) indicate extreme outliers.

**Table 1:** Comparison of OCT Measurements Between the Control and Graves’ Groups.

Parameter	Control (*n* = 60) Mean ± SD; Median (IQR)	Graves’ Group (*n* = 101) Mean ± SD; Median (IQR)	*p*-Value
CMT (µm)	268.85 ± 19.95; 265.50 (21)	269.15 ± 24.33; 267.00 (35)	0.936
cpRNFL superior (µm)	127.68 ± 17.96; 129.00 (25)	121.65 ± 18.06; 122.00 (25)	0.042
cpRNFL inferior (µm)	136.65 ± 20.01; 135.00 (25)	123.71 ± 25.87; 126.00 (34)	0.001
cpRNFL temporal (µm)	78.32 ± 8.11; 78.00 (10)	72.10 ± 12.27; 72.00 (15)	<0.001
cpRNFL nasal (µm)	85.53 ± 11.39; 82.50 (16)	81.20 ± 15.17; 81.00 (20)	0.057

Continuous variables were compared using the Mann–Whitney U test. Continuous variables are presented as mean ± standard deviation (SD) and median (interquartile range, IQR). OCT = optical coherence tomography; CMT = central macular thickness; cpRNFL = circumpapillary retinal nerve fiber layer.

**Table 2:** Comparison of systemic inflammatory indices between the control and Graves’ groups.

Inflammatory Index	Control (*n* = 60) Mean ± SD; Median (IQR)	Graves’ Group (*n* = 101) Mean ± SD; Median (IQR)	*p*-Value
NLR	2.34 ± 1.49; 1.82 (1.17)	2.27 ± 2.25; 1.85 (1.05)	0.616
PLR	145.14 ± 87.20; 125.43 (58.91)	131.27 ± 63.41; 118.13 (55.63)	0.430
MLR	0.28 ± 0.11; 0.25 (0.12)	0.29 ± 0.11; 0.28 (0.14)	0.265
SII	637.84 ± 422.25; 484.61 (431.54)	568.91 ± 474.19; 465.58 (381.88)	0.298

Continuous variables were compared using the Mann–Whitney U test. Continuous variables are presented as mean ± standard deviation (SD) and median (interquartile range, IQR). NLR = neutrophil-to-lymphocyte ratio; PLR = platelet-to-lymphocyte ratio; MLR = monocyte-to-lymphocyte ratio; SII = systemic immune-inflammation index.

**Table 3:** Comparison of Laboratory Parameters Between the Control and Graves’ Groups.

Parameter	Control (*n* = 60) Mean ± SD; Median (IQR)	Graves’ Group (*n* = 101) Mean ± SD; Median (IQR)	*p*-Value
TSH (mIU/L)	1.78 ± 0.80; 1.61 (1.12)	0.97 ± 1.21; 0.42 (1.68)	<0.001
fT3 (pg/mL)	3.26 ± 0.42; 3.25 (0.53)	3.94 ± 1.87; 3.40 (1.03)	0.006
fT4 (ng/dL)	0.85 ± 0.11; 0.85 (0.15)	1.14 ± 0.63; 0.91 (0.42)	0.001
WBC (×10⁹/L)	7.15 ± 2.11; 6.70 (3.00)	7.40 ± 2.46; 6.90 (2.05)	0.505
Hemoglobin (g/dL)	14.55 ± 1.93; 14.65 (3.10)	14.31 ± 1.61; 14.10 (2.40)	0.410
Platelet (×10⁹/L)	271.82 ± 57.06; 267.50 (62)	257.93 ± 68.39; 252.00 (82)	0.188
Neutrophil (×10⁹/L)	4.46 ± 1.72; 4.15 (2.26)	4.31 ± 1.76; 3.99 (1.78)	0.601
Lymphocyte (×10⁹/L)	2.19 ± 0.72; 2.11 (0.99)	2.22 ± 0.83; 2.11 (0.84)	0.788
Monocyte (×10⁹/L)	0.55 ± 0.16; 0.55 (0.25)	0.60 ± 0.21; 0.57 (0.25)	0.130
Glucose (mg/dL)	91.52 ± 10.42; 91.00 (12)	95.13 ± 12.35; 95.00 (18)	0.059
AST (U/L)	21.12 ± 4.56; 21.00 (7)	20.43 ± 5.90; 20.00 (7)	0.437
ALT (U/L)	20.15 ± 10.89; 16.50 (9)	19.42 ± 9.07; 18.00 (10)	0.646
Urea (mg/dL)	23.91 ± 7.19; 23.50 (12)	28.88 ± 9.80; 27.00 (11)	0.001
Creatinine (mg/dL)	0.73 ± 0.18; 0.71 (0.21)	0.73 ± 0.25; 0.71 (0.28)	0.893
Total cholesterol (mg/dL)	170.28 ± 37.63; 169.00 (50)	180.50 ± 40.80; 181.00 (59)	0.116
HDL (mg/dL)	49.12 ± 9.61; 48.50 (14)	51.22 ± 10.98; 49.00 (12)	0.221
LDL (mg/dL)	105.48 ± 31.52; 99.50 (42)	111.46 ± 37.86; 108.00 (50)	0.305
Triglyceride (mg/dL)	112.70 ± 52.84; 100.50 (46)	125.54 ± 71.55; 110.00 (53)	0.229
CRP (mg/L)	1.86 ± 1.11; 1.56 (1.13)	3.87 ± 3.40; 3.02 (2.93)	<0.001
Anti-Tg (IU/mL)	0.91 ± 0.05; 0.90 (0.0)	25.41 ± 107.12; 2.00 (5.1)	0.079
Anti-TPO (IU/mL)	0.77 ± 0.31; 0.80 (0.4)	118.22 ± 245.34; 12.40 (99.5)	<0.001

Continuous variables were compared using the Mann–Whitney U test. Continuous variables are presented as mean ± standard deviation (SD) and median (interquartile range, IQR). TSH = thyroid-stimulating hormone; fT3 = free triiodothyronine; fT4 = free thyroxine; WBC = white blood cell; AST = aspartate aminotransferase; ALT = alanine aminotransferase; HDL = high-density lipoprotein; LDL = low-density lipoprotein; CRP = C-reactive protein; Anti-TPO = anti-thyroid peroxidase antibody; Anti-Tg = anti-thyroglobulin antibody.

**Table 4:** Comparison of CMT and sectoral cpRNFL thickness among the control, GO+, and GO− groups with FDR-adjusted significance levels.

Parameter	Control Mean ± SD; Median (IQR)	GO+ Mean ± SD; Median (IQR)	GO− Mean ± SD; Median (IQR)	*p*-Value	FDR-Adjusted *q*-Value
CMT (µm)	268.85 ± 19.95; 265.50 (21)	268.26 ±26.12; 267.50 (42)	270.02 ± 22.67; 266.00 (32)	0.925	0.925
cpRNFL superior (µm)	127.68 ± 17.96; 129.00 (25)	125.72 ±18.22; 126.50 (30)	117.67 ± 17.15; 117.00 (22)	0.010	0.0125
cpRNFL inferior (µm)	136.65 ± 20.01; 135.00 (25)	131.18 ±21.90; 133.00 (27)	116.39 ± 27.52; 112.00 (43)	<0.001	0.0024
cpRNFL temporal (µm)	78.32 ± 8.11; 78.00 (10)	70.94 ± 12.15; 71.00 (16)	73.24 ± 12.40; 74.00 (14)	0.002	0.0050
cpRNFL nasal (µm)	85.53 ± 11.39; 82.50 (16)	84.76 ± 14.30; 83.00 (19)	77.71 ± 15.33; 76.00 (19)	0.006	0.0092

Continuous variables were compared using the Kruskal–Wallis test. To account for multiplicity across the five primary OCT outcomes, the Benjamini–Hochberg procedure was applied to the five omnibus Kruskal–Wallis tests to control the false discovery rate (FDR), with *q* < 0.05 considered statistically significant. For OCT outcomes remaining significant after FDR correction, pairwise comparisons were performed using Dunn’s test with Bonferroni correction. Significant pairwise differences were as follows: Superior cpRNFL: Control > GO−; Inferior cpRNFL: Control > GO−, GO+ > GO−; Temporal cpRNFL: Control > GO+, Control > GO−; Nasal cpRNFL: Control > GO−, GO+ > GO−. Continuous variables are presented as mean ± standard deviation (SD) and median (interquartile range, IQR). GO+ = Graves’ disease patients with ophthalmopathy; GO− = Graves’ disease patients without ophthalmopathy; OCT = optical coherence tomography; CMT = central macular thickness; cpRNFL = circumpapillary retinal nerve fiber layer.

**Table 5:** Comparison of systemic inflammatory indices among the control, GO+, and GO− groups.

Inflammatory Index	Control Mean ± SD; Median (IQR)	GO+ Mean ± SD; Median (IQR)	GO− Mean ± SD; Median (IQR)	*p*-Value
NLR	2.34 ± 1.49; 1.82 (1.17)	2.00 ± 0.84; 1.77 (0.96)	2.55 ± 3.05; 1.88 (1.38)	0.795
PLR	145.14 ± 87.20; 125.4 (58.91)	120.24 ± 39.49; 118.76 (58.02)	142.09 ± 79.19; 117.7 (55.55)	0.476
MLR	0.28 ± 0.11; 0.25 (0.12)	0.27 ± 0.09; 0.24 (0.13)	0.32 ± 0.12; 0.31 (0.14)	0.034
SII	637.84 ± 422.25;484.6 (431.5)	522.10 ± 247.65; 457.1 (352.81)	614.81 ± 620.7; 476.3 (500.0)	0.570

Continuous variables were compared using the Kruskal–Wallis test, followed by Dunn’s test with Bonferroni correction for post hoc pairwise comparisons where applicable. For MLR, the exploratory overall Kruskal–Wallis test yielded a nominal *p* value of 0.034; however, none of the pairwise post hoc comparisons remained statistically significant after Bonferroni correction. Continuous variables are presented as mean ± standard deviation (SD) and median (interquartile range, IQR). GO+ = Graves’ disease patients with ophthalmopathy; GO− = Graves’ disease patients without ophthalmopathy; NLR = neutrophil-to-lymphocyte ratio; PLR = platelet-to-lymphocyte ratio; MLR = monocyte-to-lymphocyte ratio; SII = systemic immune-inflammation index.

**Table 6:** Comparison of laboratory parameters among the control, GO+, and GO− groups.

Parameter	Control Mean ± SD; Median (IQR)	GO+ Mean ± SD; Median (IQR)	GO− Mean ± SD; Median (IQR)	*p*-Value
TSH (mIU/L)	1.78 ± 0.80; 1.61 (1.12)	0.93 ± 1.26; 0.44 (1.50)	1.02 ± 1.18; 0.42 (1.74)	<0.001
fT3 (pg/mL)	3.26 ± 0.42; 3.25 (0.53)	4.34 ± 2.42; 3.44 (1.17)	3.55 ± 0.97; 3.37 (0.56)	0.001
fT4 (ng/dL)	0.85 ± 0.11; 0.85 (0.15)	1.21 ± 0.79; 0.99 (0.47)	1.06 ± 0.41; 0.90 (0.32)	0.001
WBC (×10⁹/L)	7.15 ± 2.11; 6.70 (3.00)	7.54 ± 1.96; 7.25 (2.55)	7.27 ± 2.88; 6.80 (2.10)	0.677
Hemoglobin (g/dL)	14.55 ± 1.93; 14.65 (3.10)	14.48 ± 1.62; 14.20 (2.20)	14.15 ± 1.60; 13.80 (2.40)	0.461
Platelet (×10⁹/L)	271.82 ± 57.06; 267.5 (62)	265.32 ± 72.58; 248.5 (83)	250.69 ± 63.91; 253.0 (91)	0.220
Neutrophil (×10⁹/L)	4.46 ± 1.72; 4.15 (2.26)	4.40 ± 1.48; 4.08 (1.52)	4.22 ± 2.02; 3.72 (1.61)	0.763
Lymphocyte (×10⁹/L)	2.19 ± 0.72; 2.11 (0.99)	2.35 ± 0.76; 2.12 (0.73)	2.10 ± 0.89; 2.10 (0.97)	0.275
Monocyte (×10⁹/L)	0.55 ± 0.16; 0.55 (0.25)	0.59 ± 0.19; 0.56 (0.27)	0.61 ± 0.23; 0.57 (0.19)	0.298
Glucose (mg/dL)	91.52 ± 10.42; 91.00 (12)	92.56 ± 13.32; 91.50 (15)	97.65 ± 10.87; 97.00 (22)	0.015
AST (U/L)	21.12 ± 4.56; 21.00 (7)	19.36 ± 5.37; 19.00 (7)	21.47 ± 6.26; 20.00 (9)	0.109
ALT (U/L)	20.15 ± 10.89; 16.50 (9)	19.38 ± 10.35; 17.50 (10)	19.45 ± 7.73; 18.00 (11)	0.900
Urea (mg/dL)	23.91 ± 7.19; 23.50 (12)	27.79 ± 10.52; 26.50 (10)	29.94 ± 9.01; 28.00 (12)	0.002
Creatinine (mg/dL)	0.73 ± 0.18; 0.71 (0.21)	0.71 ± 0.20; 0.68 (0.28)	0.75 ± 0.30; 0.74 (0.31)	0.648
Total cholesterol (mg/dL)	170.28 ± 37.63; 169.0 (50)	177.48 ± 37.95; 172.0 (51)	183.47 ± 43.59; 194.0 (61)	0.219
HDL (mg/dL)	49.12 ± 9.61; 48.50 (14)	48.64 ± 9.49; 46.50 (9)	53.75 ± 11.81; 52.00 (11)	0.023
LDL (mg/dL)	105.48 ± 31.52; 99.50 (42)	108.36 ± 40.31; 102.0 (37)	114.49 ± 35.42; 114.0 (47)	0.408
Triglyceride (mg/dL)	112.70 ± 52.84; 100.5 (46)	137.50 ± 87.02; 123.0 (74)	113.82 ± 50.34; 101.0 (43)	0.091
CRP (mg/L)	1.86 ± 1.11; 1.56 (1.13)	3.22 ± 3.14; 2.41 (2.96)	4.51 ± 3.56; 3.48 (2.89)	<0.001
Anti-Tg (IU/mL)	0.91 ± 0.05; 0.90 (0.0)	44.46 ± 149.54; 3.60 (25.1)	6.74 ± 17.83; 0.90 (1.6)	0.017
Anti-TPO (IU/mL)	0.77 ± 0.31; 0.80 (0.4)	159.22 ± 274.6; 22.6 (207.2)	78.03 ± 207.63; 4.80 (21.8)	<0.001

Continuous variables were compared using the Kruskal–Wallis test, followed by Dunn’s test with Bonferroni correction for post hoc pairwise comparisons where applicable. The *p*-values presented in the p-value column represent the omnibus Kruskal–Wallis tests, whereas *p*-values reported in the Results text for specific pairwise comparisons represent Bonferroni-corrected Dunn’s post hoc tests. Because these laboratory comparisons were exploratory, the omnibus *p*-values are reported as nominal *p*-values. Significant post hoc pairwise differences after Bonferroni correction were as follows: TSH: Control > GO+, GO−; fT3: GO+ > Control, GO−; fT4: GO+ > Control; Glucose: GO− > Control; Urea: GO− > Control; HDL: GO− > GO+; CRP: GO+, GO− > Control; Anti-Tg: GO+ > Control; Anti-TPO: GO+ > Control. Continuous variables are presented as mean ± SD and median (IQR). GO+ = Graves’ disease patients with ophthalmopathy; GO− = Graves’ disease patients without ophthalmopathy; TSH = thyroid-stimulating hormone; WBC = white blood cell count; AST = aspartate aminotransferase; ALT = alanine aminotransferase; HDL = high-density lipoprotein; LDL = low-density lipoprotein; CRP = C-reactive protein; Anti-Tg = anti-thyroglobulin antibody; Anti-TPO = anti-thyroid peroxidase antibody.

**Table 7:** Spearman correlation analysis between laboratory parameters, age, and OCT measurements with Benjamini–Hochberg FDR correction.

Variable	OCT Parameter	*r*	Raw p	FDR-Adjusted *p* (*q*)
TSH	CMT	−0.094	0.236	0.5829
	Superior cpRNFL	−0.031	0.699	0.8738
	Inferior cpRNFL	−0.005	0.952	0.9811
	Temporal cpRNFL	0.133	0.094	0.5153
	Nasal cpRNFL	0.052	0.515	0.7831
fT3	CMT	0.037	0.643	0.8281
	Superior cpRNFL	−0.076	0.335	0.6723
	Inferior cpRNFL	−0.001	0.994	0.9940
	Temporal cpRNFL	−0.035	0.663	0.8411
	Nasal cpRNFL	0.004	0.958	0.9811
fT4	CMT	0.074	0.348	0.6723
	Superior cpRNFL	−0.174	0.027	0.5153
	Inferior cpRNFL	−0.076	0.341	0.6723
	Temporal cpRNFL	−0.093	0.239	0.5829
	Nasal cpRNFL	−0.137	0.084	0.5153
WBC	CMT	−0.049	0.535	0.7831
	Superior cpRNFL	0.111	0.160	0.5364
	Inferior cpRNFL	0.019	0.811	0.9443
	Temporal cpRNFL	0.120	0.129	0.5364
	Nasal cpRNFL	0.038	0.630	0.8238
Neutrophil	CMT	−0.049	0.537	0.7831
	Superior cpRNFL	0.135	0.088	0.5153
	Inferior cpRNFL	0.089	0.263	0.6042
	Temporal cpRNFL	0.080	0.310	0.6588
	Nasal cpRNFL	0.115	0.147	0.5364
Lymphocyte	CMT	−0.021	0.789	0.9443
	Superior cpRNFL	−0.006	0.939	0.9811
	Inferior cpRNFL	−0.067	0.400	0.7083
	Temporal cpRNFL	0.043	0.588	0.7933
	Nasal cpRNFL	−0.103	0.195	0.5525
Monocyte	CMT	0.017	0.835	0.9591
	Superior cpRNFL	−0.046	0.562	0.7831
	Inferior cpRNFL	−0.072	0.365	0.6894
	Temporal cpRNFL	−0.091	0.251	0.5926
	Nasal cpRNFL	0.002	0.975	0.9866
CRP	CMT	0.019	0.810	0.9443
	Superior cpRNFL	−0.004	0.957	0.9811
	Inferior cpRNFL	−0.157	0.047	0.5153
	Temporal cpRNFL	−0.068	0.395	0.7083
	Nasal cpRNFL	−0.141	0.075	0.5153
Platelet	CMT	−0.078	0.323	0.6696
	Superior cpRNFL	0.050	0.527	0.7831
	Inferior cpRNFL	0.039	0.626	0.8238
	Temporal cpRNFL	0.119	0.132	0.5364
	Nasal cpRNFL	−0.012	0.878	0.9725
Triglyceride	CMT	−0.144	0.069	0.5153
	Superior cpRNFL	−0.094	0.235	0.5829
	Inferior cpRNFL	−0.132	0.094	0.5153
	Temporal cpRNFL	−0.118	0.136	0.5364
	Nasal cpRNFL	−0.051	0.523	0.7831
Anti-Tg	CMT	0.082	0.300	0.6538
	Superior cpRNFL	−0.010	0.904	0.9791
	Inferior cpRNFL	0.020	0.801	0.9443
	Temporal cpRNFL	−0.109	0.169	0.5364
	Nasal cpRNFL	−0.046	0.561	0.7831
Anti-TPO	CMT	−0.055	0.492	0.7831
	Superior cpRNFL	−0.142	0.073	0.5153
	Inferior cpRNFL	−0.058	0.469	0.7666
	Temporal cpRNFL	−0.163	0.038	0.5153
	Nasal cpRNFL	−0.147	0.063	0.5153
Age	CMT	−0.085	0.286	0.6397
	Superior cpRNFL	−0.044	0.583	0.7933
	Inferior cpRNFL	−0.174	0.028	0.5153
	Temporal cpRNFL	−0.127	0.110	0.5364
	Nasal cpRNFL	−0.145	0.066	0.5153

**Note:** *r*, Spearman’s rank correlation coefficient; CMT, central macular thickness; cpRNFL, circumpapillary retinal nerve fiber layer; TSH, thyroid-stimulating hormone; fT3, free triiodothyronine; fT4, free thyroxine; WBC, white blood cell count; CRP, C-reactive protein; anti-Tg, anti-thyroglobulin antibody; anti-TPO, anti-thyroid peroxidase antibody. Raw *p* values are two-sided. To account for multiple testing, the Benjamini–Hochberg false discovery rate (FDR) procedure was applied jointly across the 85 correlation tests reported in Table 7 and Table 8. FDR-adjusted *p* values (*q* values) < 0.05 were considered statistically significant. Bold raw *p* values indicate associations that reached nominal significance (*p* < 0.05) before FDR correction; none remained statistically significant after FDR correction.

**Table 8:** Spearman correlation analysis between systemic inflammatory indices and OCT measurements with Benjamini–Hochberg FDR correction.

Variable	OCT Parameter	*r*	Raw *p*	FDR-Adjusted *p* (*q*)
NLR	CMT	−0.046	0.560	0.7831
	Superior cpRNFL	0.093	0.240	0.5829
	Inferior cpRNFL	0.107	0.175	0.5364
	Temporal cpRNFL	0.061	0.441	0.7617
	Nasal cpRNFL	0.156	0.049	0.5153
PLR	CMT	−0.013	0.869	0.9725
	Superior cpRNFL	0.068	0.393	0.7083
	Inferior cpRNFL	0.106	0.183	0.5364
	Temporal cpRNFL	0.059	0.457	0.7617
	Nasal cpRNFL	0.115	0.147	0.5364
MLR	CMT	0.009	0.910	0.9791
	Superior cpRNFL	0.012	0.881	0.9725
	Inferior cpRNFL	0.019	0.807	0.9443
	Temporal cpRNFL	−0.105	0.183	0.5364
	Nasal cpRNFL	0.113	0.152	0.5364
SII	CMT	−0.060	0.453	0.7617
	Superior cpRNFL	0.131	0.097	0.5153
	Inferior cpRNFL	0.096	0.224	0.5829
	Temporal cpRNFL	0.112	0.158	0.5364
	Nasal cpRNFL	0.142	0.072	0.5153

**Note:** *r*, Spearman’s rank correlation coefficient; CMT, central macular thickness; cpRNFL, circumpapillary retinal nerve fiber layer; NLR, neutrophil-to-lymphocyte ratio; PLR, platelet-to-lymphocyte ratio; MLR, monocyte-to-lymphocyte ratio; SII, systemic immune-inflammation index. Raw *p* values are two-sided. To account for multiple testing, the Benjamini–Hochberg false discovery rate (FDR) procedure was applied jointly across the 85 correlation tests reported in Table 7 and Table 8. FDR-adjusted *p* values (*q* values) < 0.05 were considered statistically significant. Bold raw *p* values indicate associations that reached nominal significance (*p* < 0.05) before FDR correction; none remained statistically significant after FDR correction.

**Table 9:** Multivariable linear regression analysis of factors associated with inferior cpRNFL thickness among patients with Graves’ disease (*n* = 101).

Predictor	B	SE	β	t	p	95% CI for B	Tolerance	VIF
GO+ vs. GO−	12.926	5.085	0.251	2.542	0.013	2.831 to 23.020	0.939	1.065
Age	−0.340	0.255	−0.132	−1.337	0.184	−0.846 to 0.165	0.940	1.064
Male vs. female	0.289	5.150	0.006	0.056	0.955	−9.933 to 10.511	0.917	1.090
ln(anti-TPO + 1)	1.544	1.302	0.124	1.187	0.238	−1.039 to 4.128	0.842	1.188

Model statistics: *R² = 0.121; adjusted R² = 0.084; F(4,96) = 3.294; p = 0.014.* **Note:** B, unstandardized regression coefficient; SE, standard error; β, standardized regression coefficient; CI, confidence interval; VIF, variance inflation factor; GO+, Graves’ disease with ophthalmopathy; GO−, Graves’ disease without ophthalmopathy; cpRNFL, circumpapillary retinal nerve fiber layer; anti-TPO, anti-thyroid peroxidase antibody. Ophthalmopathy status was coded as 0 = GO− and 1 = GO+, and sex as 0 = female and 1 = male. Because of marked right-skewness, anti-TPO was entered into the model as ln(anti-TPO + 1). The dependent variable was inferior cpRNFL thickness.

## Data Availability

The datasets generated and/or analyzed during the current study are available from the corresponding author upon reasonable request.

## Abbreviations

The following abbreviations are used in this manuscript:

OCT	Optical coherence tomography
RNFL	Retinal nerve fiber layer
GO	Graves’ ophthalmopathy
GO+	Graves’ disease with ophthalmopathy
GO−	Graves’ disease without ophthalmopathy
TSH	Thyroid-stimulating hormone
fT3	Free triiodothyronine
fT4	Free thyroxine
Anti-TPO	Anti-thyroid peroxidase antibody
Anti-Tg	Anti-thyroglobulin antibody
CRP	C-reactive protein
NLR	Neutrophil-to-lymphocyte ratio
PLR	Platelet-to-lymphocyte ratio
MLR	Monocyte-to-lymphocyte ratio
SII	Systemic immune-inflammation index
CBC	Complete blood count
CMT	Central macular thickness
SD	Standard deviation
IQR	Interquartile range
